# Brain asymmetry and its association with inattention and heritability during neurodevelopment

**DOI:** 10.1038/s41398-025-03327-1

**Published:** 2025-03-26

**Authors:** Dardo Tomasi, Nora D. Volkow

**Affiliations:** National Institute on Alcohol Abuse and Alcoholism, Bethesda, MD USA

**Keywords:** Predictive markers, Epigenetics and behaviour

## Abstract

The relationship between brain asymmetry and inattention, and their heritability is not well understood. Utilizing advanced neuroimaging, we examined brain asymmetry with data from the Adolescent Brain Cognitive Development (ABCD; n = 8943; 9–10 y) and the Human Connectome Project (HCP) cohorts (n = 1033; 5–100 y). Data-driven metrics from resting-state fMRI and morphometrics revealed reproducible and stable brain asymmetry patterns across the lifespan. In children, high levels of inattention were highly heritable (61%) and linked to reduced leftward asymmetry of functional connectivity in the dorsal posterior superior temporal sulcus (dpSTS), a region interconnected with a left-lateralized language network. However, reduced dpSTS asymmetry had low heritability (16%) and was associated with lower cognitive performance suggesting that non-genetic factors, such as those mediating cognitive performance, might underlie its association with dpSTS asymmetry. Interventions that enhance cognition might help optimize brain function and reduce inattention.

## Introduction

Among the myriad facets of brain organization, asymmetry—the uneven distribution of functions and structures between the brain’s hemispheres—has garnered considerable attention for its role in shaping cognitive and emotional processes [1–5]. This asymmetry manifests not only in the lateralization of language [6] and motor [7] functions but also in the nuanced differences observed across various cognitive and emotional domains, including attention [8] and emotion processing [9]. Despite its significance, the relationship between brain asymmetry, cognitive traits, and their heritability remains poorly understood. Within this broader exploration of brain asymmetry, here we focus on the superior temporal sulcus as a key example due to its established role in left-lateralized language and social cognition networks, and its observed links to cognitive performance and inattention.

Attention, a cognitive process vital for regulating behavior and facilitating information processing, is a multifaceted construct involving several neural networks distributed across the brain [10]. One region implicated in attentional processes is the posterior part of the superior temporal sulcus (pSTS), situated along the lateral surface of the temporal lobe. The pSTS is a complex and functionally diverse structure known for its role in social perception and processing [11]. The pSTS is also involved in various aspects of social attention [11–13], including the perception of biological motion [14], interpretation of facial expressions [12], and inference of others’ mental states—a process known as theory of mind [15]. Additionally, the posterior pSTS integrates sensory information from different modalities, such as vision and audition, to facilitate the perception and interpretation of socially relevant stimuli [16]. Notably, the engagement of pSTS in attentional processes extends beyond social perception [17], with emerging evidence suggesting its involvement in directing attention toward salient environmental stimuli, including speech and other socially relevant cues [18, 19].

While the precise mechanisms underlying pSTS asymmetry remain incompletely understood [20, 21], converging evidence suggests that this asymmetry plays a crucial role in shaping human cognition and behavior [22]. Studies investigating pSTS asymmetry have revealed consistent leftward biases in brain activation during language and social cognition tasks [23]. For instance, fMRI studies have consistently reported left-lateralized activations within the pSTS during tasks involving speech perception [24]. Additionally, the left hemisphere dominance of the pSTS has been implicated in the perception and interpretation of social cues [25].

Sex differences in left-hemispheric dominance and language development have been controversial [26]. Prior fMRI studies have shown that women show more bilateral patterns of activity during language tasks than men [27–29]. While meta-analyses did not find significant effects of sex on language lateralization [30] it is possible that differences in lateralization emerge when focusing specifically on complex story comprehension [31].

Resting-state functional connectivity has been used to measure functional asymmetry in language areas, including the inferior frontal (Broca’s area) and superior temporal cortices in the brain of healthy adults [32]. Further, the trajectory of interhemispheric functional asymmetry in language-related regions, based on resting-state brain images obtained during the first 2 years of life, predicted language outcomes at 4 years of age [33].

In this study, we aim to extend our understanding of pSTS asymmetry and its implications for attentional processes in children. By leveraging advanced neuroimaging techniques and large-scale longitudinal data from the Adolescent Brain Cognitive Development (ABCD) study and the Human Connectome Project (HCP), we seek to investigate the consistency of asymmetry patterns in brain morphometry and functional connectivity. Additionally, we aim to explore the impact of brain development, genetic influences, and shared environmental factors on these asymmetry patterns, with a specific focus on understanding their associations with sex and inattention in 9–10-year-old US children. We used data-driven metrics of brain activity and functional connectivity derived from resting-state fMRI scans, as well as cortical thickness and sulcal depth morphometrics extracted from MRI structural scans taking advantage of the large cohort from the ABCD study. We hypothesized that children with higher inattention scores would exhibit reduced asymmetry in pSTS compared to children with lower scores. Since women had greater structural asymmetry in the STS and other language regions compared to men [34], we also hypothesized that girls would exhibit greater asymmetry in language-related regions compared to boys.

## Methods

### Participants

The ABCD study monitors over 11,800 children into adulthood with annual lab tests and biannual MRI scans. Participants without medical/cognitive issues, limited English proficiency, or MRI safety concerns were included [35]. Local institutional review boards (IRB) at the University of California in San Diego and 21 data collection sites across the United States approved the ABCD study [36]. The study participants mirrored the demographics of the US population [37]. We examined brain scans and behavior data from 9521 children at baseline in the ABCD study (2.0 data release) [38] with resting-state fMRI data in CIFTI format. In statistical analysis, we excluded 560 participants with excessive levels of head motion during resting-state fMRI (>50% of time points with framewise displacement, FD < 0.5 mm), as well as 8 participants without inattention scores, and 10 participants with undefined race. Thus, the final sample for studies on brain asymmetry included 8943 children (Table 1). For external validation of brain asymmetry metrics, we analyzed neuroimaging data of 450 adolescents (14.3 ± 3.8 years old; 211 males and 239 females) from the Development cohort of the Human Connectome Project (HCP-D; https://www.humanconnectome.org/study/hcp-lifespan-development) [39] and 583 adults (57.3 ± 14.1 years old; 249 males and 334 females) from the Aging HCP cohort (HCP-A; https://www.humanconnectome.org/study/hcp-lifespan-aging) [40] with available resting-state fMRI data in CIFTI format.

**Table 1:** Characteristics of the *Discovery*, *Replication*, and *Normality* samples.

	Discovery	Replication	Normality	P
Sample size	4395	4326	222	
Sex (F/M)	2176/2219	2062/2264	110/112	0.22^Ϯ^
Age [years]	9.94 (0.62)	9.94 (0.62)	9.89 (0.63)	0.73*
White	2434	2377	114	0.89^Ϯ^
Black	589	578	35	
Hispanic	849	832	40	
Asian	81	80	5	
Other	442	459	28	
Inattention score	2.82 (3.39)	2.82 (3.41)	3.12 (3.58)	0.99*
Brain volume [mL]	1208 (112)	1213 (114)	1209 (106)	0.03*
Framewise displacement [mm]	117 (41)	116 (42)	122 (42)	0.14*
Siemens	2862	2920	150	0.11^Ϯ^
GE	1026	911	45	
Phillips	507	495	27	
Fluid composite score	92.1 (10.3)	92.2 (10.4)	91.3 (10.7)	0.67*
Crystalized composite score	86.7 (6.7)	86.7 (6.9)	86.7 (7.6)	0.90*
Total composite score	86.8 (8.7)	86.8 (8.9)	86.2 (9.4)	0.89*

p: 2-sided statistical differences between the *Discovery* and *Replication* samples using 2-sample t-test* or χ^2^-test^Ϯ^.

### Inattention

We downloaded the attention problems sub-scale of the Child Behavior Checklist (CBCL) from the NIMH data archive (NDA: https://nda.nih.gov). The CBCL contains 10 items, which were rated by parents on a 3-point Likert scale (0 = not at all true; 1 = somewhat true; 2 = very true) and summarize attention problems: 1-acts too young for his/her age; 2-fails to finish things he/she started; 3-can’t concentrate, can’t pay attention for long; 4-can’t sit still, restless or hyperactive; 5-confused or seems to be in a fog; 6-daydreams or gets lost in his/her thoughts; 7-impulsive or acts without thinking; 8-poor schoolwork; 9-inattentive or easily distracted; and 10-stares blankly. The inattention score was equated to the summary of the scores on these 10- items and treated as a continuous variable (0–20).

### Cognitive performance

The ABCD research team calculated fluid composite scores using participant’s performance scores on pattern comparison processing speed, list-sorting working memory, picture sequence memory, Flanker, and dimensional change card sort tests, and crystalized composite scores using the scores on oral reading recognition and picture vocabulary tests. Total cognitive composite scores were derived from fluid and crystalized composite scores [41].

### Family income

The ABCD study data on annual household income was obtained from NDA.

### MRI data

The ABCD imaging procedures were standardized for 3T MRI scanners (Siemens Prisma, Phillips, and General Electric 750 scanners) that were equipped with adult-sized multi-channel coils and capable of performing multiband echo planar imaging (EPI). These procedures were implemented across 21 sites, and further details can be found elsewhere [42, 43]. Briefly, structural MRI employed 3D T1w inversion-prepared RF-spoiled gradient echo and T2w variable flip angle fast spin echo pulse sequences with 1 mm isotropic resolution. Functional MRI (fMRI) data were acquired using T2*-weighted multiband echo planar imaging (EPI) with parameters including TE/TR of 30/800 ms, 2.4 mm isotropic resolution, a flip angle of 52 degrees, 60 slices covering the entire brain, and a multiband slice acceleration of 6 [42]. For our analysis of brain asymmetry, we relied on the ABCD brain imaging data structure Community Collection (ABCC; https://collection3165.readthedocs.io/en/stable/), which includes high quality fMRI data, acquired in resting-state conditions over 20 min, and brain morphometrics (cortical thickness and sulcal depth) from over 10,000 children [44]. The HCP-A and HCP-D imaging procedures are similar to those of the ABCD study and are described elsewhere [45]. Briefly, 3 T Siemens Prisma scanners were used for MRI employing T1w MPRAGE [46] and T2w SPACE [47] sequences with 0.8 mm isotropic resolution for structural imaging, and T2*-weighted multiband EPI (TE/TR of 37/800 ms, 2 mm isotropic resolution, a flip angle of 52 degrees, 72 slices covering the entire brain, and MB acceleration of 8) was used for fMRI data acquisition [45]. For our analysis of brain asymmetry, we relied on the CIFTI datasets with minimal image preprocessing provided by the HCP [48].

### Reproducibility

We used the ABCC’s “matched group” status, which is based on sociodemographic factors that can impact brain development (age, sex, ethnicity, grade, highest level of parental education, handedness) [44] to split ABCD participants into 3 independent demographically matched subsamples: *Discovery* (N = 4395, girls = 2176), *Replication* (N = 4326, girls = 2062), and *Normality* (N = 222; girls = 110).

### ABCD-BIDS pipeline

Like the HCP pipeline, the ABCD-BIDS pipeline comprises 5 consecutive steps: *PreFreesurfer*, performs brain extraction, denoising, and normalization of structural data to a standard template; *Freesurfer*, performs brain segmentation and creates cerebral surfaces with FreeSurfer [43], which has been validated for use in children [49]; *PostFreesurfer*, converts brain surfaces into the HCP-compatible CIFTI format; *fMRIVolume*, registers the functional time series to the volumetric standard template; and *fMRISurface*, converts functional time series data to the CIFTI format. Differences between the HCP and ABCD-BIDS pipelines are fully described elsewhere [44]. Briefly, the ABCD-BIDS pipeline does not require T2w images and performs the nonlinear registration to the standard atlas in *PostFreeSurfer*, which increases the effectiveness of the registration. Additionally, the ABCD-BIDS pipeline uses ANTS [50] for nonlinear registration which consistently outperforms other nonlinear registration methods [51]. In addition, the *fMRISurface* step in the ABCD-BIDS pipeline includes functional connectivity pre-processing that separates true head motion from fictitious motion induced by breathing-related magnetic field changes [52], and performs standard denoising by regressing out time-varying head motion, white matter and CSF signals, and the global signals that may impact group comparisons [53, 54], from both dense (dtseries) and parcellated (ptseries) CIFTI datasets within the 360 cortical partitions [55] and the 19 subcortical partitions obtained from Freesurfer (HCP2016FreeSurferSubcortical_dparc.dlabel.nii), which is also included in the data release of the ABCC.

### Head motion

Motion-censoring data, determined using the ABCD-BIDS pipeline, was utilized to eliminate time frames with frame-wise displacement (FD) > 0.5 mm.

### fALFF and gFCD

We used the fractional amplitude of low-frequency fluctuations (fALFF), a marker of brain activity [56], to quantify the proportion of spontaneous signal fluctuations in 0.01–0.1 Hz band [57]. The global functional connectivity density (gFCD) [58] was used to quantify degree. Specifically, for each gray ordinate we calculated the total number of edges that exceed a Pearson correlation > 0.6 [58, 59], and its logarithm was equated to gFCD. fALFF and gFCD were mapped from individual time series with N = 91,282 grayordinates [48] and a maximum of 1520 time points (20 min) using Matlab 2022a (MathWorks, inc., Natick, MA) and the Biowulf cluster at NIH (https://hpc.nih.gov/).

### dpSTS functional connectivity

We used gFCD-guided seed correlation analyses to explore the functional connectivity patterns of the dorsal posterior part of the superior temporal sulcus (dpSTS) cluster in which inattention was linked to decreased gFCD asymmetry. Specifically, the connectome workbench function cifti-average-roi-correlation was used to map the functional connectivity of a bilateral dpSTS seed (center vertex# 9165, 10-mm radius) from individual motion-corrected and 0.01–0.10 Hz band-pass filtered time series in CIFTI space.

### Brain asymmetry index

A normalized index of asymmetry contrasted intensity values in the left (LH) and right (RH) hemispheric surfaces, independently for each metric (gFCD, fALFF, cortical thickness, and sulcal depth) and individual:

1 $$\Delta =\frac{{metric}\left({LH}\right)-{metric}\left({RH}\right)}{|{metric}\left({LH}\right)|+|{right}\left({RH}\right)|}$$

To confirm the correspondence of 32,492 vertices in the LH with those in the RH, using correlation analysis of the Cartesian coordinates of every vertex, relative to the bounding box center (xyz = 180 mm, 218 mm, 180 mm). The interhemispheric correspondence of vertices in LH and RH was better than 99% (R > 0.995; Supplementary Fig S1) [7].

### Genetic contribution to imaging and behavioral phenotypes

We downloaded ABCD genetic information (gen_y_pihat.csv) from NDA and identified 304 monozygotic (MZ) twin pairs and 464 dizygotic (DZ) twin pairs. Pairwise correlations for brain asymmetry (sulcal depth, cortical thickness, gFCD, and fALFF) and behavioral (fluid, crystalized, total composite, and inattention scores) traits were calculated within MZ and DZ pairs. Prior to analysis, the trait data were adjusted for the effects of age using linear regression and those of sex, race, scanner manufacturer, and research site with grand mean scaling, and the residuals were used in subsequent modeling to minimize variance attributable to these confounding factors. The heritability of the trait was estimated using structural equation modeling within the OpenMx package in R (https://cran.r-project.org/web/packages/OpenMx/index.html) assuming that genetic contributions to the trait variance are shared 100% by MZ twins and 50% by DZ twins. An ACE model with 4 parameters was specified, which decomposes the variance of the trait into additive genetic (A), shared environmental (C), and unique environmental (E) components. The model included paths from latent variables A, C, and E to the corrected traits. Model fit was assessed using Akaike (AIC) and Bayesian (BIC) information criteria. The parameter estimates for the genetic (A), shared environmental (C), and unique environmental (E) components were examined to determine the heritability of the trait. Standard errors and confidence intervals (CI) for these estimates were calculated to assess their precision.

### ROI analysis

Average asymmetry values within each of the 180 partitions of the multi-modal parcellation of the left cerebral cortex [55], were independently computed for each individual to assess the associations of the asymmetry metrics with inattention.

### Statistical analyses

We confirmed the normal distribution of the asymmetry metrics within the dpSTS multi-modal ROI in the independent *Normality* subsample using the Shapiro–Wilk normality test [60] (W > 0.99; p > 0.09). Then, we regressed out effects of head motion and brain volume, independently across boys and girls, and removed unwanted effects associated with age and race from the asymmetry metrics. To assess the main effect of inattention on asymmetry metrics we conducted a factorial analysis of covariance (ANCOVA) in MATLAB using sex as a categorical covariate, independently for the *Discovery* and *Replication* subsamples. We also investigated the main effects of sex and inattention and their interaction on brain asymmetry while controlling for the effects of age, brain volume, cognitive performance, family income, scanner manufacturer, and remaining mean FD, using type 3 ANCOVA in R with the package rstatix (https://www.rdocumentation.org/packages/rstatix/versions/0.7.2/topics/anova_test). A false discovery rate threshold pFDR<0.05 was used to correct for multiple comparisons across 91,282 grayordinates or 180 ROIs. To adjust p-values for tests of spatial correlation we used the spin method randomization technique [61] with 100,000 random spin permutations.

## Results

### Characteristics of the ABCD sample

**Fig. 1:**
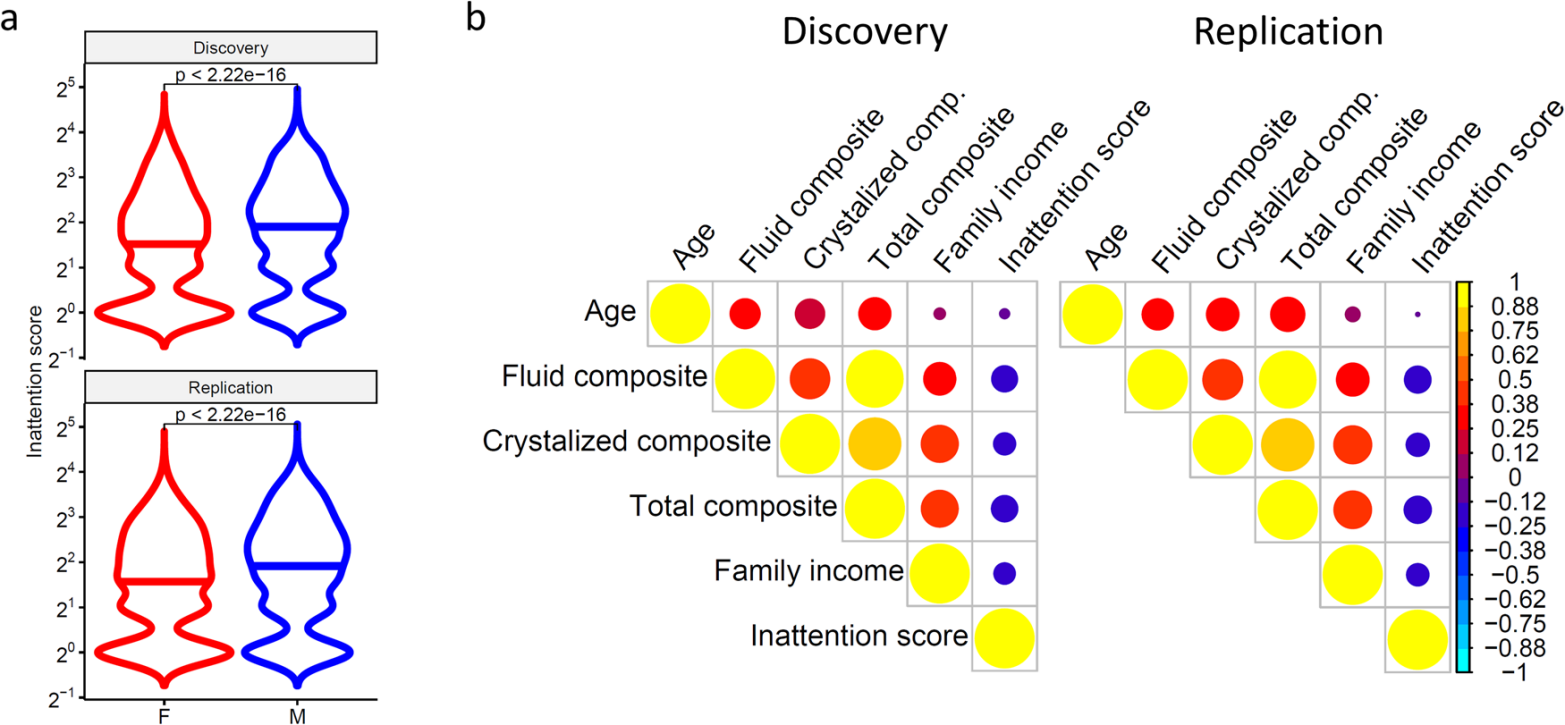
Inattention. Violin plots showing the reproducibility of the distribution of inattention scores across boys (M) and girls (F) in Discovery and Replication subsamples **a**. Reproducibility of the Pearson correlation matrix linking inattention, family income, chronological age, and fluid, crystalized, and total cognitive composite scores in Discovery and Replication subsamples **b**. Sample size: 4348 girls and 4595 boys from the ABCD study.

Among the 8943 children in the present study, 8222 (92%) had inattention scores<9 and 721 (8%) had inattention scores ≥ 9 (Table 1); note that the inattention 9/20 threshold cutoff predicted the diagnosis of attention-deficit-hyperactivity disorder (ADHD) [62]. These proportions align with the rate of US children and adolescents aged 2–17 years with a parent-reported ADHD diagnosis (9.4%) in the 2017–2018 period (https://www.cdc.gov/ncbddd/adhd/timeline.html), which corresponds to the time frame when the ABCD baseline data was collected. Analysis of sex differences revealed a higher inattention score for boys (3.33 ± 3.67) than girls (2.89 ± 3.01; t(8775) = 14.8, P < 2.2E-16, 2-sided t-test; Fig. 1a), which is consistent with the sex differences in the prevalence of ADHD among US children and adolescents (https://www.cdc.gov/nchs/data/databriefs/db499.pdf). Moreover, inattention scores had weak but significant negative correlations with family income and cognitive performance (fluid, crystalized, and total composite scores; R < −0.3; P < 2.2E-16; Fig. 1b).

### Discovery and replication subsamples

There were no statistical differences in inattention scores, sex proportion, age, race, in-scanner head motion (framewise displacement), scanner manufacturer, and cognitive performance between Discovery and Replication subsamples (P > 0.11; Table 1). The effects of sex, race, and cognitive performance on the inattention score in Fig. 1 reproduced in Discovery and Replication subsamples (Fig S2).

### Brain asymmetry

**Fig. 2:**
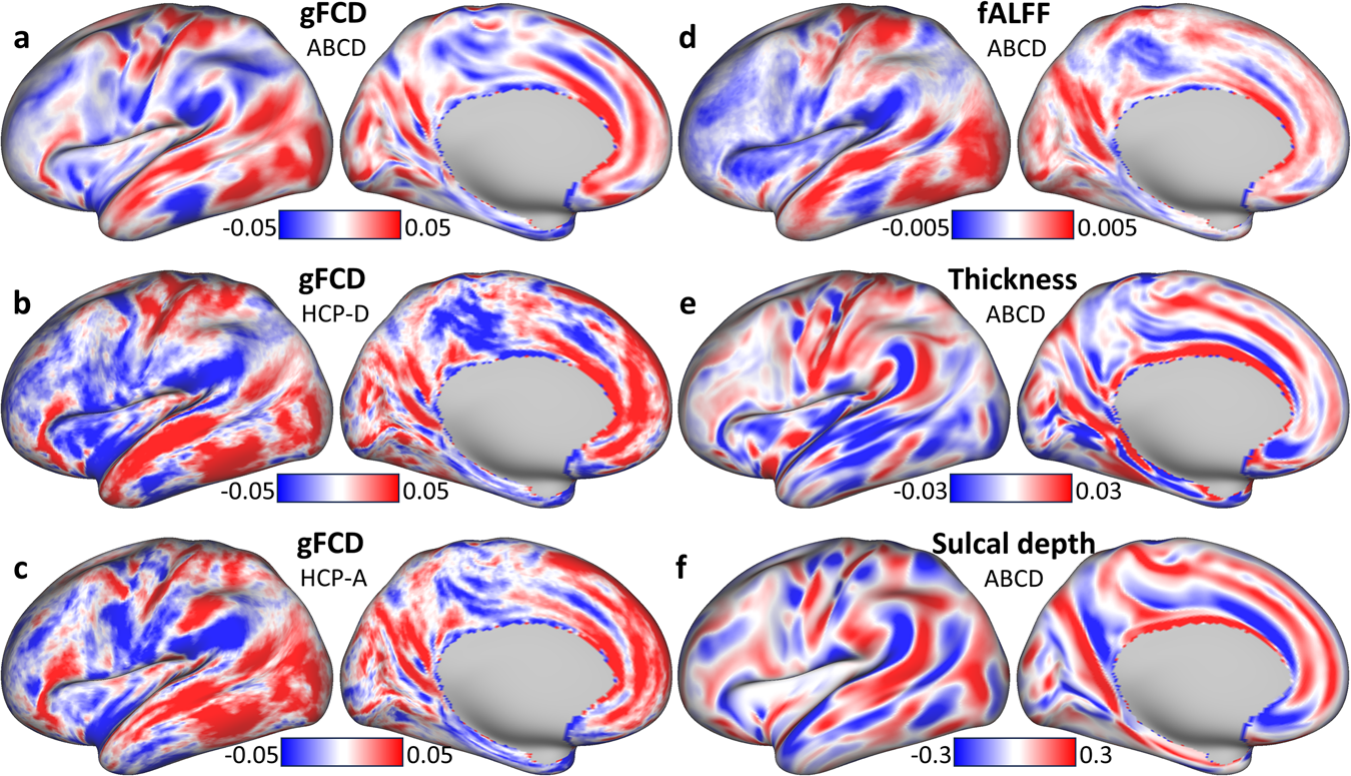
Asymmetry index. Average asymmetry index, Δ, for global functional connectivity density (gFCD; **a**–**c**, fractional amplitude of low-frequency fluctuations (fALFF; **d**, cortical thickness **e**, and sulcal depth **f** for 8721 children (ABCD), 450 adolescents (HCP-D), and 583 adults (HCP-A), superimposed on lateral and medial views of the left cerebral hemisphere. Red and blue colors indicate leftward and rightward asymmetry, respectively.

To investigate brain asymmetry, we used a normalized index of asymmetry, Δ, contrasting corresponding values of a metric in the left and right hemispheric surfaces [7]. Specifically, we subtracted values on the right hemisphere from the corresponding ones on the left hemisphere, such that Δ > 0 indicated leftward asymmetry, and Δ > 0 rightward asymmetry. This allowed us to map Δ across the 32,492 vertices on the left cortical hemisphere (Fig. 2). With Δ we investigated resting-state functional asymmetry using global functional connectivity density (gFCD), a marker of hubness [59], and fractional amplitude of low-frequency fluctuations (fALFF), a marker of brain activity [56]. In addition, with Δ we studied the asymmetry of cortical thickness and sulcal depth morphometrics estimated from MRI structural scans using FreeSurfer (https://surfer.nmr.mgh.harvard.edu/).

The leftward gFCD asymmetry predominated in STS, visual and auditory cortices, somatomotor and medial default-mode network (DMN) regions, and language areas (Fig. 2a). This asymmetry pattern reflects the dominance of the left hemisphere in functions related to visual and auditory processing, motor control, language function, and aspects of self-referential cognition. The rightward gFCD asymmetry predominated in inferior parietal and dorsolateral prefrontal (DLPFC) cortices, and insular-opercular regions. This asymmetry pattern reflects the dominance of the right hemisphere in functions related to attentional control, certain executive functions, sensorimotor integration, and interoceptive and emotional processing. These asymmetry patterns were highly reproducible in Discovery and Replication subsamples of the ABCD cohort (Fig S3) as well as in the Development (HCP-D; Fig. 2b) and Aging (HCP-A; Fig. 2c) cohorts of the Human Connectome Project. Specifically, there was a high spatial correlation across 32,492 vertices between the average Δ patterns from Discovery and Replication samples (R = 0.998), as well as with those corresponding to the HCP-D (R = 0.937) and HCP-A (R = 0.939) cohorts (p_spin_ < 2E-05). The asymmetry patterns of fALFF were similar to those of gFCD (R > 0.834; p_spin_ < 5E-05; Fig. 2d) but show rightward asymmetry in Broca’s area, instead of leftward asymmetry. The asymmetry patterns of gFCD were weakly anticorrelated with those of cortical thickness and sulcal depth (R < -0.225; p_spin_ < 5E-05; Fig. 2e, f). For fALFF, cortical thickness, and sulcal depth measures, the reproducibility of asymmetry in Discovery and Replication subsamples was also very high (R > 0.998; p_spin_ < 1E-05; Fig S3).

### Development and asymmetry

Due to the narrow age range in the ABCD cohort, we combined the ABCD cohort with the HCP-D cohort to investigate the variability of brain asymmetry as a function of age from childhood to adulthood.

The effects of age in asymmetry were most prominent for fALFF whereas they were minimal for gFCD, cortical thickness, and sulcal depth (Fig S4). In the case of fALFF, regions that showed predominant rightward asymmetry tended to decrease with age (i.e., DLPFC, inferior parietal cortex) whereas regions that showed leftward asymmetry tended to increase with age (i.e., somatomotor, STS). The asymmetry of fALFF in STS, somatomotor and other cortical regions changed with age differently in boys and girls (P_FDR_ < 0.05; Fig S4). A subsequent whole-brain analysis, employing the root-mean square of asymmetry, revealed age-by-sex interaction effects. These effects indicated that the absolute asymmetries of fALFF and gFCD decreased with age more significantly in boys than in girls (Fig S5).

### Effect of sex

**Fig. 3:**
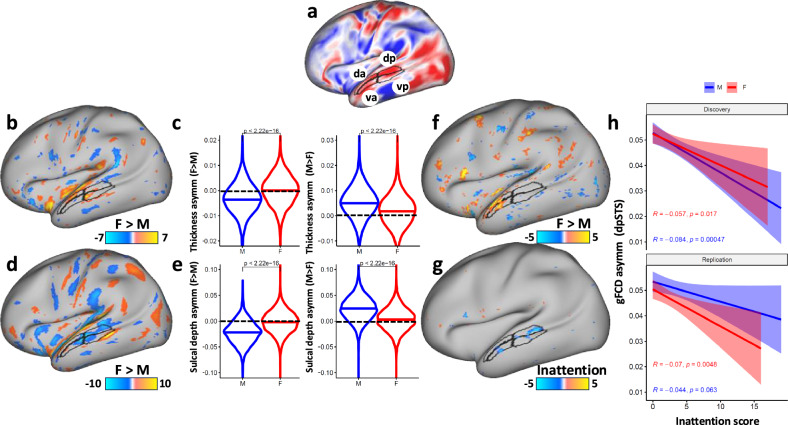
Effects of sex and inattention on brain asymmetry. **a** Leftward (red) and rightward (blue) asymmetry for global functional connectivity density (gFCD), superimposed on the lateral cerebral surface, highlighting the leftward gFCD asymmetry in dorsal anterior (da), dorsal posterior (dp), ventral anterior (va), and ventral posterior (vp) regions of the superior temporal sulcus (STS; black contours; multi-modal parcellation of the left cerebral cortex [55]). Statistical difference (t-score) in the asymmetry of cortical thickness **b** and sulcal depth **d** between 4238 girls **F** and 4483 boys (M) from the ABCD study, overlaid on a lateral view of the left cortical hemisphere. Violin plots **c** and **e** highlight the boy’s higher leftward lateralization, averaged in all regions where F > M (red pattern in b and d), and their higher rightward lateralization, averaged in all regions where M > F (blue pattern in b and d). **f**, **g** ANCOVA T-score maps showing the higher leftward asymmetry of gFCD in the anterior STS ROIs for girls than boys (f), and the association between higher inattention and lower leftward gFCD asymmetry in STS (g). The negative association between inattention and gFCD asymmetry in dpSTS reproduced in boys and girls, and in the Discovery and Replication subsamples **h**; numeric labels reflect 2-sided Pearson correlations. a PFDR < 0.05 threshold was used for display.

Cortical thickness and sulcal depth asymmetries differed between boys and girls in several ROIs (Fig. 3b–e; Supplementary Tables S1 and S2). In STS, the asymmetries of cortical thickness (rightward) and sulcal depth (leftward) were stronger for boys than girls (Fig. 3; Table S1). For gFCD, girls had higher asymmetry in dorsal (daSTS), and ventral (vaSTS) regions of the anterior STS (P_FDR_ < 0.05, Fig. 3f), and other ROIs including Broca’s area (Brodmann areas 44 and 45), middle premotor cortex (area 55b), primary auditory cortex, the insula, and inferior parietal and DLPFC regions (Table S3). The effect of sex on fALFF asymmetry was restricted to the dorsal part of the pSTS (dpSTS), parietal, and visual areas (Fig S6; Table S4).

### Effect of inattention

Higher inattention scores were linked to lower leftward asymmetry of gFCD and fALFF in STS, predominately in dpSTS (Tables S3 and S4), where the negative association with inattention reproduced in Discovery and Replication subsamples both in boys and girls (Fig. 3h), and in Brodman area 44 (P_FDR_ < 0.05, Fig. 3g). The effect of inattention on the asymmetries of cortical thickness and sulcal depth was not significant (Fig S6; Tables S1, S2, and S4).

### Handedness

To control for potential confounding of handedness in the asymmetry measures, we excluded left-handed (n = 624) and mixed handed (n = 1114) children and confirmed the effects of sex and inattention on the asymmetry metrics (Fig S7) in right-handed children (n = 7001) only.

### Associations between asymmetry metrics

Increased gFCD asymmetry in the dpSTS was associated with increased asymmetry of fALFF (R > 0.53) and to a lesser extent with asymmetry in cortical thickness, and sulcal depth in both girls and boys (R > 0.04, P < 0.017; Fig S8).

### Asymmetry of the dpSTS network

**Fig. 4:**
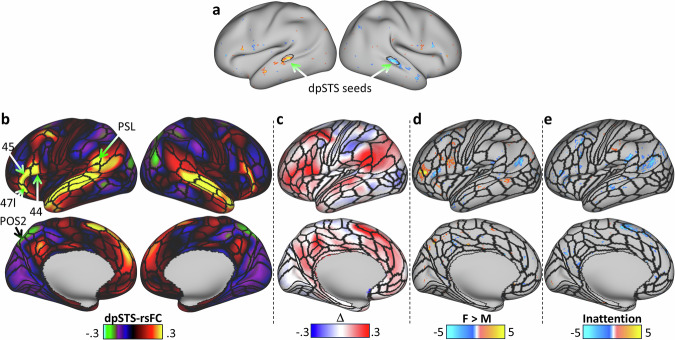
dpSTS network. **a** A bilateral seed in the dorsal posterior region of the superior temporal sulcus (dpSTS), which had lower asymmetry of global functional connectivity density with increased inattention scores, was used to map the resting-state functional connectivity (rsFC) using seed-vertex correlation analyses. **b** dpSTS connectivity mapped into classical language regions, such as the inferior frontal cortex (Brodmann areas 44, 45, and 47l) and the Peri-Sylvian language (PSL) area. **c** dpSTS-rsFC asymmetry, Δ, mapped into the left cerebral hemisphere showing the strong leftward lateralization (red) of language network connectivity in STS, PSL, and the inferior frontal cortex. T-score maps reflecting statistically significant differences in Δ between 4238 girls (F) and 4483 boys (M); **d** as well as associations with inattention scores and Δ across children **e**, overlaid on the left cerebral hemisphere using a P_FDR_ < 0.05 corrected threshold. Black lines are contours of a multi-modal parcellation of the left cerebral cortex [55]. Statistical model: ANCOVA. Data from the ABCD study. POS2 Parieto-occipital sulcus area 2.

We assessed the connectivity patterns of the bilateral dpSTS cluster that demonstrated decreased gFCD asymmetry with increased inattention with seed-vertex correlation analyses (Fig. 4a; Table S5). The functional connectivity of the dpSTS seed was high in all STS partitions, temporo-parieto-occipital junction area 1, superior temporal visual area STV, inferior parietal language areas PF and PGi, inferior (Brodmann areas 44 and 45) and superior frontal (area SFL) language regions, and medial DMN regions within Brodmann areas 9, 10, and 31 (P_FDR_ < 0.05; Fig. 4b; Table S5). In these language regions, the dpSTS-rsFC had leftward asymmetry (P_FDR_ < 0.05; Fig. 4c; Table S5). The negative functional connectivity of the dpSTS seed was strongest in the parietal cortex (parieto-occipital sulcus area 2, POS2; P_FDR_ < 0.05; Fig. 4b), which exhibited rightward asymmetry of dpSTS-rsFC (P_FDR_ < 0.05; Fig. 4c). In ROI analysis, the leftward asymmetry of dpSTS-rsFC in the inferior frontal cortex was stronger for girls than boys, and that in temporal pole and Brodmann area 9 was stronger for boys than girls (Table S5; Fig. 4d). Higher inattention scores were linked to weaker leftward asymmetry of dpSTS-rsFC in multiple ROIs, including dorsal and ventral pSTS (Table S5; Fig. 4e). We confirmed these findings in the subsample of right-handed children (Fig S9). In left-handers (273 girls and 362 boys), the asymmetry of the dpSTS-rsFC pattern matched that in the whole sample (Fig. 4c), but the effects of sex and inattention on dpSTS-rsFC asymmetry did not reach significance (Fig S10).

### Brain activation

Using the activation patterns to a language task that contrasted “story” and “math” epochs across 997 healthy young adults [55], we verified the overlap of the dpSTS network with the brain regions that engaged in language processing (Fig S11a). Specifically, compared to math epochs, story epochs activated all STS partitions, as well as areas 44, 45, 47l, 55b, PGi, SFL, TPOJ1, STV, and PSL, as well as medial DMN regions.

### Brain asymmetry and cognitive performance

Using linear regression analysis to test for potential associations between brain asymmetry in dpSTS and cognitive performance, we found positive correlations for fALFF or gFCD asymmetry with crystalized and total composite scores (R(6778) > 0.025; P < 0.04), but the correlations were not significant for fluid composite scores, which for gFCD reproduced in boys and girls (Fig S11b). Cognitive performance did not show significant association with cortical thickness of sulcal depth asymmetry. We confirmed these findings in the subsample of right-handed children (Fig S12).

### Genetic and shared environmental contributions

**Fig. 5:**
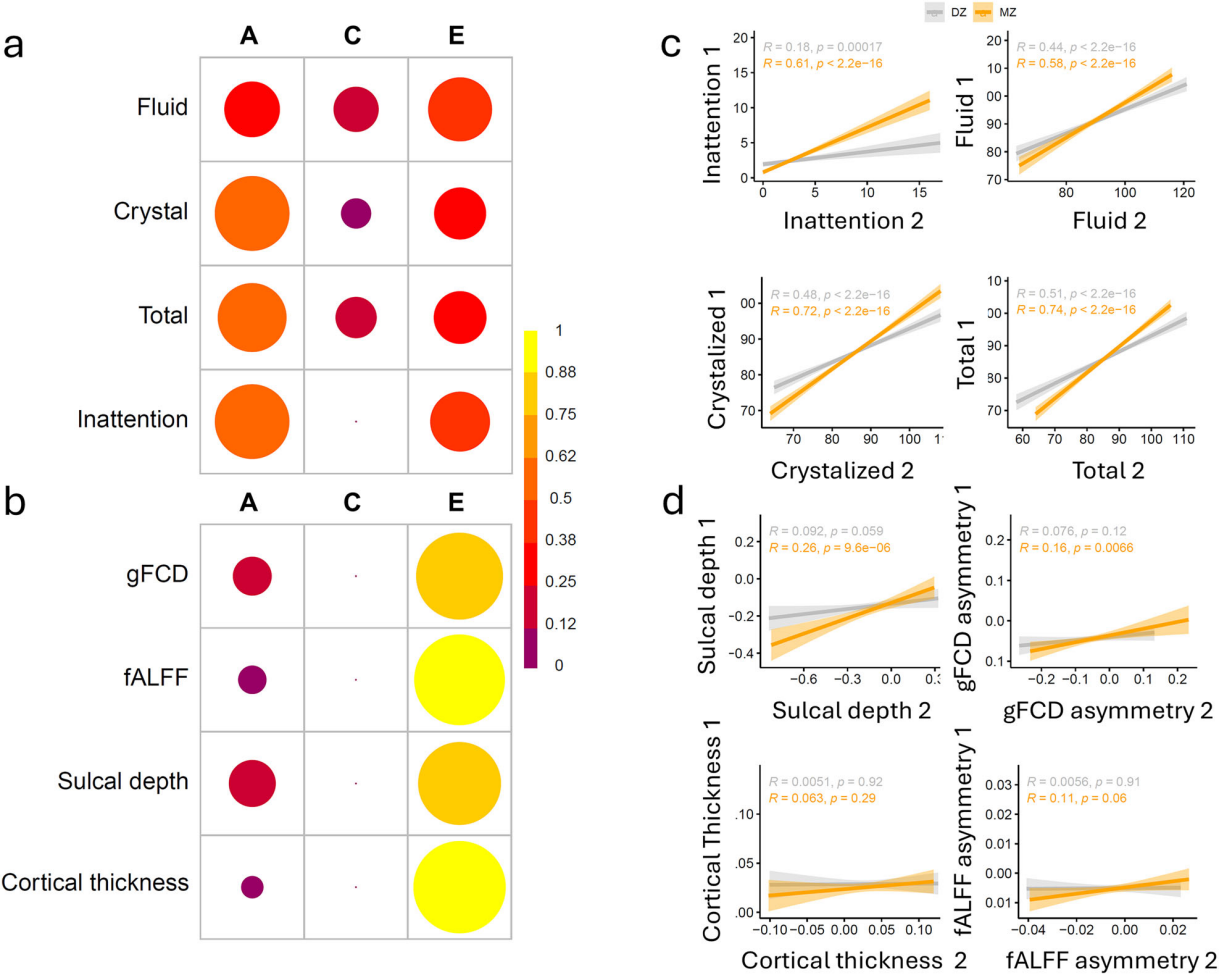
Genetic and environmental effects. Variance explained by additive genetic factors (A), shared environmental factors (C), and non-shared environmental factors (E) for behavioral metrics (inattention scores and fluid, crystalized, and total composite scores) **a** and measures of brain asymmetry (cortical thickness, sulcal depth, global functional connectivity density, gFCD, and the fractional amplitude of low-frequency fluctuations, fALFF) **b** in the dorsal posterior part of the superior temporal sulcus (dpSTS) across 304 pairs of monozygotic (MZ) twins and 464 pairs of dizygotic (DZ) twins. Pairwise correlations demonstrate that behavioral **c** and brain asymmetry **d** metrics are more similar among MZ twin pairs than among DZ twin pairs. The color scale represents the percentage of variance explained, with darker colors indicating lower percentages of variance attributed to the specific factors.

To estimate the heritability of the inattention, cognitive composites, and brain asymmetry, we used the data from 304 monozygotic (MZ) twin pairs and 424 dizygotic (DZ) twin pairs in the ABCD sample. To control for potential confounds from age, sex, race, scanner manufacturer, and research site, we removed the variability associated to these confounds before performing the estimations. The variance of the trait was decomposed into three components: additive genetic (A), shared environmental (C), and unique environmental (E). For inattention, the additive genetic component (A) accounted for 61% of the variance (95% CI: 59–63%), indicating a substantial genetic influence on the trait (Fig. 5a; Table S6). The shared environmental component (C) did not contribute significantly to the variability of inattention scores. The remaining 39% of the variance was attributed to unique environmental factors (E) (95% CI: 38–40%), which include individual-specific experiences and measurement error. For fluid, crystalized and total cognitive performance A accounted for 34, 61, and 52% of the variance, respectively, also indicating a substantial genetic influence on these traits. The shared and unique environment accounted for >39% (9% < C < 22% and 29% < E < 45%). Differently, the heritability of sulcal depth and gFCD asymmetries in dpSTS were modest (A < 24%), and those for cortical thickness and fALFF asymmetries were minimal (A < 8%; Fig. 5b; Table S6). The estimations of heritability are consistent with the higher pairwise correlations for MZ than DZ, differences that were stronger for cognitive (fluid, crystalized, total composite) and inattention scores than for sulcal depth, cortical thickness, gFCD, and fALFF traits (Fig. 5c, d). For these brain measures the influence of non-shared environmental factors accounted for the most variance in their asymmetry patterns (Fig. 5b).

## Discussion

Here using various functional and structural brain metrics we uncover brain asymmetry patterns with predominant leftward asymmetry in visual, superior temporal, somatomotor, DMN, and language areas, and predominant rightward asymmetry in the inferior parietal cortex, DLPFC, and insular-opercular regions across the lifespan. This asymmetry pattern was highly reproducible across the Discovery and Replication subsamples of the ABCD study, as well as in the HCP development and aging cohorts. The consistency of brain asymmetry patterns across these additional HCP datasets suggests that the observed asymmetry patterns generalize across individuals and different developmental and aging stages.

fALFF asymmetry in STS and other brain regions increased with age even within the narrow age range of the children (108–131 months), an effect that was more pronounced in girls than boys. This supports recent findings on changes in the asymmetry of brain activity, particularly in regions involved in auditory processing, language comprehension, and social cognition, which are functions associated with the STS, during infant development [63]. Our results also endorse the emergence of functional asymmetries in the temporal cortex during childhood and adolescence, a process that may be delayed in children with autism spectrum disorder [64]. In contrast, age-related changes in asymmetry during childhood/adolescence were significantly weaker for gFCD, cortical thickness, and sulcal depth, suggesting that asymmetry of functional connectivity and morphometry remains relatively stable during development.

Boys demonstrated stronger leftward brain asymmetry in morphometrics than girls, consistent with the notion that brain lateralization is stronger in males than females [65]. However, the sex differences in brain asymmetry had low effect sizes and were more pronounced for structural than functional metrics. The inferior frontal cortex demonstrated higher gFCD asymmetry in girls than boys, an observation consistent with the sex differences in functional segregation and integration of brain networks during language processing [66], which may reflect differential developmental trajectories of language networks between boys and girls. Overall, our findings are in line with brain maturation processes, and environmental factors, which may contribute to the establishment and refinement of language networks during development, leading to variations in connectivity patterns and asymmetry between sexes [67].

Higher levels of inattention were associated with lower asymmetry of gFCD and fALFF in dpSTS, suggesting a potential link between asymmetry disruptions in this region and attentional difficulties. Disrupted connectivity asymmetry in dpSTS, a region implicated in auditory processing and social cognition [11–14, 16] that is important for directing attention toward salient environmental stimuli [18, 19], could lead to deficits in selective attention and cognitive performance. For instance, selective attention to phonetic and emotional stimuli have been shown to elicit strong left-lateralized hemodynamic responses in pSTS in humans [68]. Furthermore, attention deficits have been linked to lower fMRI signals within the STS in macaques [69], and lower STS volume have been reported in children with ADHD [70]. Also, deficits in attention and higher order biological motion processing were associated to lower STS volume in schizophrenia patients [71], and theta-burst stimulation have been shown to change the functional connectivity of the STS and improve attention to the neglected hemisphere in stroke patients [72]. However, in boys, the correlation between inattention and gFCD asymmetry in dpSTS was significant in the Discovery subsample but did not fully replicate at the 0.05 threshold in the Replication subsample (p = 0.063). In contrast, for girls the correlation was significant in both the Discovery and Replication subsamples. This discrepancy highlights potential sex differences in the relationship between inattention and asymmetry, which warrants further investigation in larger and more balanced datasets to clarify these patterns.

The bilateral dpSTS seed had strong functional connectivity of leftward asymmetry in all language network regions, consistent with the lateralization of language [73]. The association between increased gFCD asymmetry in dpSTS with higher dpSTS connectivity in Brodmann area 44, which is involved in speech production, further supports the involvement of dpSTS in language-related processes [23, 24]. The observed association suggests a coordinated functional relationship between dpSTS and the inferior frontal cortex.

The lack of significant effects of inattention or sex on dpSTS asymmetry for left-handers, in contrast to the significant effects observed in right-handers, may partly reflect limitations in statistical power due to the smaller sample size of left-handers (n = 635) compared to right-handers (n = 7001). Left-handers often exhibit greater heterogeneity in functional asymmetries, which could further dilute potential sex effects [74]. Thus, the absence of significant effects of sex and inattention does not necessarily indicate a lack of biological or developmental differences but rather suggests that larger or more targeted datasets may be required to detect such effects reliably.

Both fALFF and gFCD asymmetry in dpSTS showed positive associations with cognitive performance across multiple domains in both boys and girls. In contrast, neither cortical thickness nor sulcal depth asymmetry in dpSTS showed significant correlations with cognitive performance. These findings are consistent with the notion that brain functional lateralization affects behavior/performance across species [75], and highlight the importance of dpSTS asymmetry in supporting cognitive functions related to fluid, crystallized, and overall cognitive abilities.

The findings from the ACE model based on MZ and DZ comparisons from the ABCD data set showed high heritability of inattention scores (A = 61%), underscoring a significant genetic influence on inattention scores. This genetic influence is similar to that for the heritability of inattentiveness and hyperactivity in twin studies of children/adolescents with ADHD (74%) [76]. However it is notably higher than the heritability estimate for inattention scores (h^2^_SNP_ = 20%) based on 3563 children/adolescents derived from the proportion of phenotypic variance explained by the aggregate effects of single nucleotide polymorphisms (SNPs) across the genome [77]. Similarly, the ACE model showed significantly higher heritability for cognitive abilities (34% < A < 61%) than that reported based on SNP heritability (13%< h^2^_SNP_ < 25%) [77]. The higher heritability estimates that we obtained from the ACE model compared to that from previous estimated based on polygenic risk scores is consistent with the discordant heritability values reported for other complex traits [78]. The discrepancies reflect a gap of broader genetic influence, encompassing all genetic variance in the twin studies that GWAS studies have been unable to capture with common SNPs. The heritability estimates for brain asymmetry were small with only a modestly higher concordance across MZ than DZ in morphometric asymmetry in dpSTS. This is consistent with prior reports of heritability for brain structural asymmetry [4, 79], and those in gFCD and fALFF asymmetries in dpSTS (<23%), which suggest that brain asymmetry metrics are strongly influenced by environmental factors with much less of an effect from direct genetic influences. Our findings suggest that while brain asymmetry is associated with certain behaviors, it may not be the primary driver of these traits. Instead, asymmetry might act as one of many intermediate neural phenotypes influenced by broader genetic and environmental factors [80], while traits might emerge from complex networks of interactions that are only partially reflected in asymmetry patterns.

We observed a strong positive linear association between gFCD and fALFF asymmetry patterns, suggesting concurrent asymmetries in connectivity and spontaneous brain activity, consistent with the notion that higher functional connectivity is associated with higher brain metabolism and spontaneous activity [81, 82]. Notably, asymmetry patterns in fALFF and gFCD were largely similar, except for the inferior frontal cortex (areas 44 and 45), which exhibited leftward gFCD asymmetry and rightward fALFF asymmetry. This may indicate a more complex interplay between hemispheric specialization and language processing in the inferior frontal cortex.

Considering the dpSTS’s link to the language network, activities that engage and stimulate language processing, such as reading, storytelling, and language games, could potentially enhance leftward asymmetry. Similarly, exercises aimed at improving attention span and focus, like computer-based attention training programs, mindfulness, and meditation, might also help enhance dpSTS asymmetry and reduce inattention. Use of neuromodulation, such as intermittent theta burst stimulation (iTBS) [83], to boost the left dpSTS and enhance leftward asymmetry might also help reduce inattention. Future studies could leverage data from the ABCD Study to explore whether engagement in language-related activities is associated with greater leftward asymmetry in language-processing regions. The ABCD dataset includes detailed assessments of participants’ daily activities, including the type and frequency of games played, time spent reading, and other educational or recreational pursuits that could provide valuable insights into the extent to which environmental factors shape functional asymmetry during development.

Our study has limitations, including the narrow age range of children in the ABCD dataset, which may restrict the generalizability of our findings to other stages of brain development. However, the longitudinal design of the ABCD study allows for future investigations to examine whether changes in brain asymmetry over time correlate with improvements in attention and cognitive performance as neurodevelopment progresses.

Our findings have implications for our understanding of the origins and plasticity of brain asymmetry and underscore the need for future research to explore the complex interplay between genetic, environmental, and developmental factors in shaping brain organization.

## Supplementary information

Supplementary Methods and Results

## Data Availability

ABCD data are publicly available through the National Institute of Mental Health Data Archive (https://data-archive.nimh.nih.gov/abcd).
