# Supplementary material for: Brain asymmetry and its association with inattention and heritability during neurodevelopment
Source: Transl Psychiatry. 2025 Mar 26;15:96. doi: 10.1038/s41398-025-03327-1 (PMC11947263; doi:10.1038/s41398-025-03327-1)
Supplement: Supplementary file 1 — Supplementary Methods and Results [file 41398_2025_3327_MOESM1_ESM.docx]

Supplementary material for:

**Brain Asymmetry and its Association with Inattention and Heritability during Neurodevelopment**

Dardo Tomasi*^1^ and Nora D. Volkow^1^

^1^National Institute on Alcohol Abuse and Alcoholism, Bethesda, MD, 20892

**Recruitment.** Information regarding recruitment methods and criteria for the ABCD study can be found in published materials accessible on the ABCD website (https://abcdstudy.org/scientists/protocols/). In short, the ABCD study employed probability-based sampling of U.S. schools located within 21 catchment areas, which are geographical regions centered around schools within a 50-mile radius of the research institution, as the primary approach for identifying and enrolling eligible children and their parents [1]. Families were given recruitment materials and digital copies. Families expressing interest underwent a short telephone screening. If eligible, they were enrolled and scheduled for the baseline assessment at the research centers. Both guardians and children received compensation for their participation [1]. Recruitment closely represented demographic variables (sex, race, ethnicity, parental marital status and education, and income) of the general US population [2].

**Inclusion and Exclusion criteria**: Children were included unless they had severe psychiatric or neurological disorders or significant medical conditions. Exclusion criteria comprised common MRI contraindications, limited fluency in English, uncorrected sensory impairments, major neurological disorders in their medical history, very premature birth (<28 weeks), extremely low birth weight (<1,200 g), prolonged hospitalization due to birth complications, current diagnosis of schizophrenia, moderate to severe autism spectrum disorder, a history of traumatic brain injury, or unwillingness to participate in assessments [3, 4]. Children, whether taking medications or not, were considered for inclusion.

**fALFF.** The preprocessed fMRI data were processed to compute the fractional amplitude of low-frequency fluctuations (fALFF) in CIFTI space [5]. Specifically, the fast Fourier transform was used to compute the power spectrum of the fMRI signal at each grayordinate x0, and fALFF(x0) was defined as the power within the 0.01-0.10 Hz low-frequency band at x0, normalized by the total power across all frequencies in the entire spectrum at x0. The fALFF patterns were highly reproducible in the Discovery and Replication subsamples (Fig S2). The strongest spontaneous signal fluctuations were observed in the posterior precuneus, angular gyrus, visual areas, and dorsolateral and inferior PFC.

**gFCD.** Two grayordinates were deemed functionally connected when their time-varying signals exhibited a correlation greater than 0.6. This specific correlation threshold was chosen to align with the threshold applied in FCD mapping [6]. The gFCD (also called degree) at a given grayordinate, x0, was determined as the natural logarithm of the total number of functional connections (edges in the adjacency matrix) k(x0), between x0 and all other grayordinates throughout the brain. This calculation was applied to all grayordinates in the brain, resulting in the computation of a correlation matrix with 91,282x91,282 elements. The estimation of gFCD was carried out using Matlab 2017b (MathWorks, Inc., Natick, MA). The averaged gFCD pattern were highly reproducible across children in Discovery and Replication subsamples (Fig S3) and exhibited similarities to that of adults [7]. Specifically, robust connectivity hubs were observed in the posterior cingulate cortex (PCC), occipital, motor, and inferior parietal regions, which play crucial roles in organizing resting-state networks (RSN)[8]. Notably, subcortical regions exhibited relatively lower gFCD, except for hubs located in the posterior cerebellar lobe.

**Graphical tools.** The Connectome Workbench ([https://www.humanconnectome.org](https://www.humanconnectome.org/software/connectome-workbench)) and RStudio (<https://www.rstudio.com/>) were used for the visualization of CIFTI data and to create the figures.

**
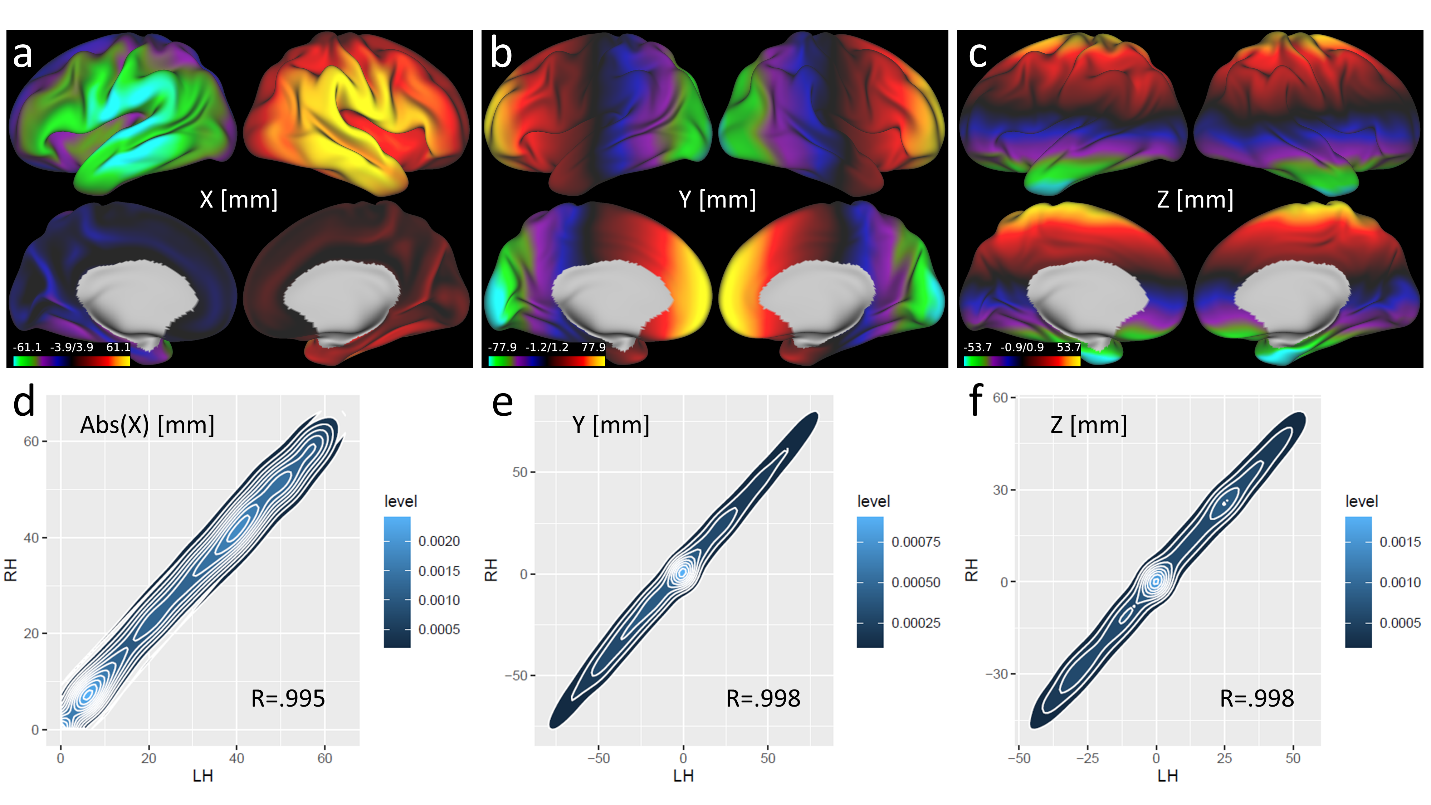
**

**Fig. S1: Interhemispheric vertex correspondence.** Cartesian coordinates of 64,894 vertices on the cerebral surface (**a**-**c**). 2d-density plots showing the correspondence of these coordinates in the left (LH) and right (RH) hemispheres (**d-f**).


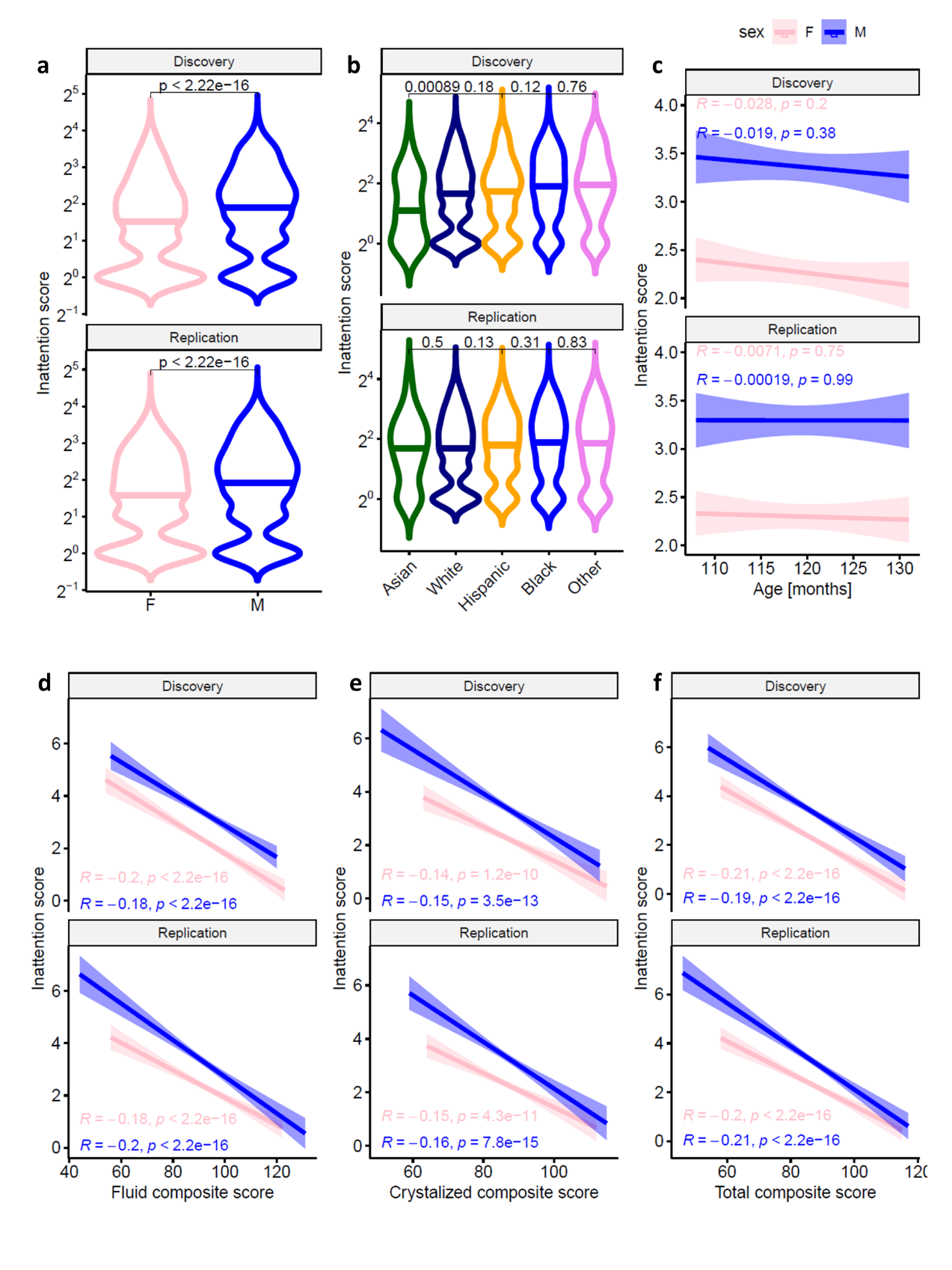


**Fig S2. Inattention:** Reproducibility in Discovery and Replication subsamples of the distribution of inattention scores across 4,348 girls (F) and 4,595 boys (M) (**a**), 166 Asian, 4925 White, 1721 Hispanic, 1202 Black, and 929 children of mixed race (Other) (**b**). Reproducibility of the associations of the inattention score with chronological age (**c**), and fluid, crystalized, and total cognitive composite scores (**d-f**). Numeric labels in violin plots are p-values for 2-sided t-test comparisons (a-b). R- and p-values in scatter plots are Pearson correlation factors.


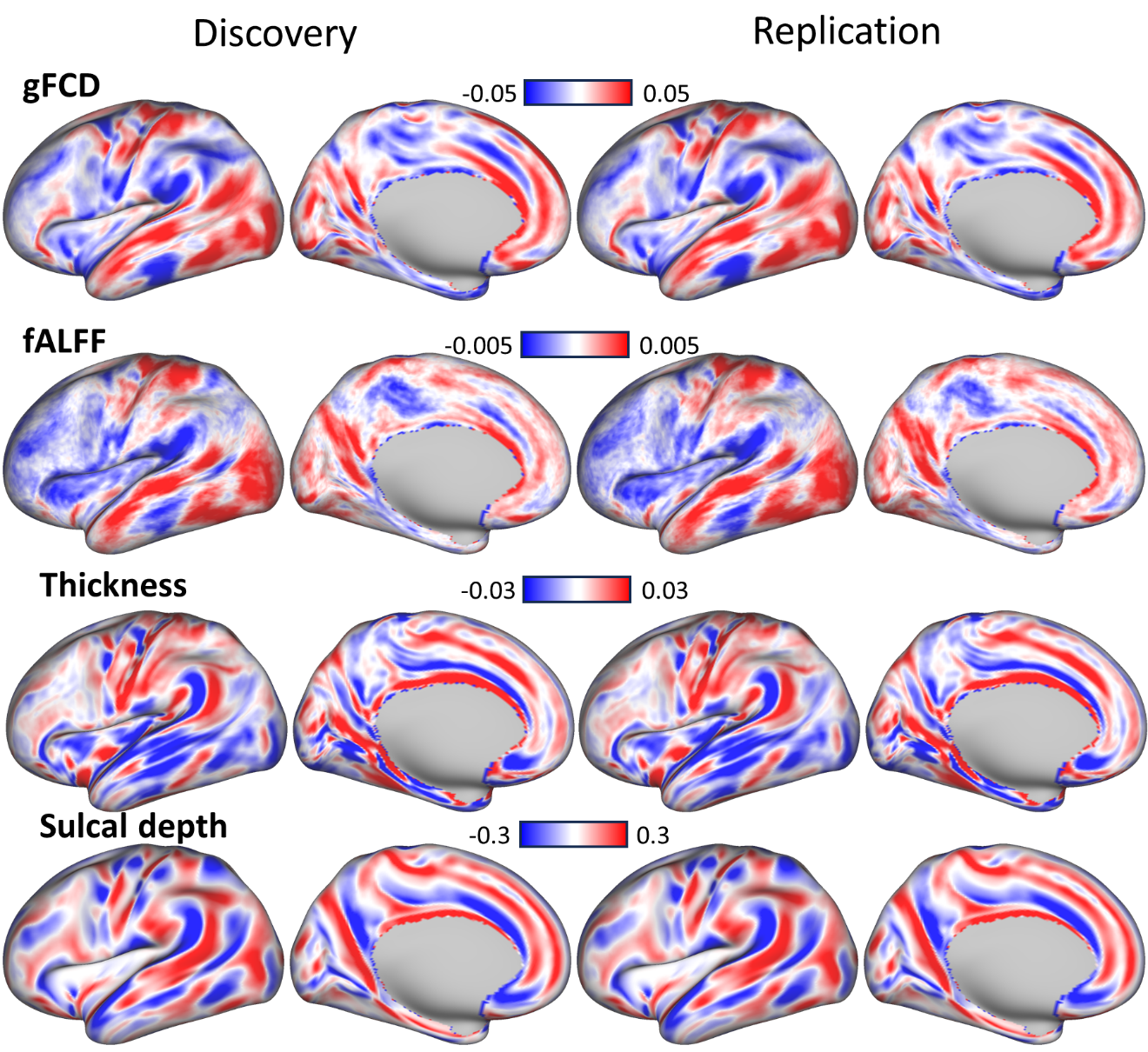


**Fig S3. Asymmetry index.** Reproducibility in Discovery (n=4,395 children) and Replication (n=4,326 children) subsamples of the average asymmetry index, Δ, for global functional connectivity density (gFCD), fractional amplitude of low-frequency fluctuations (fALFF), cortical thickness, and sulcal depth, superimposed on lateral and medial views of the left cerebral hemisphere. Red and blue colors indicate leftward and rightward lateralization, respectively.


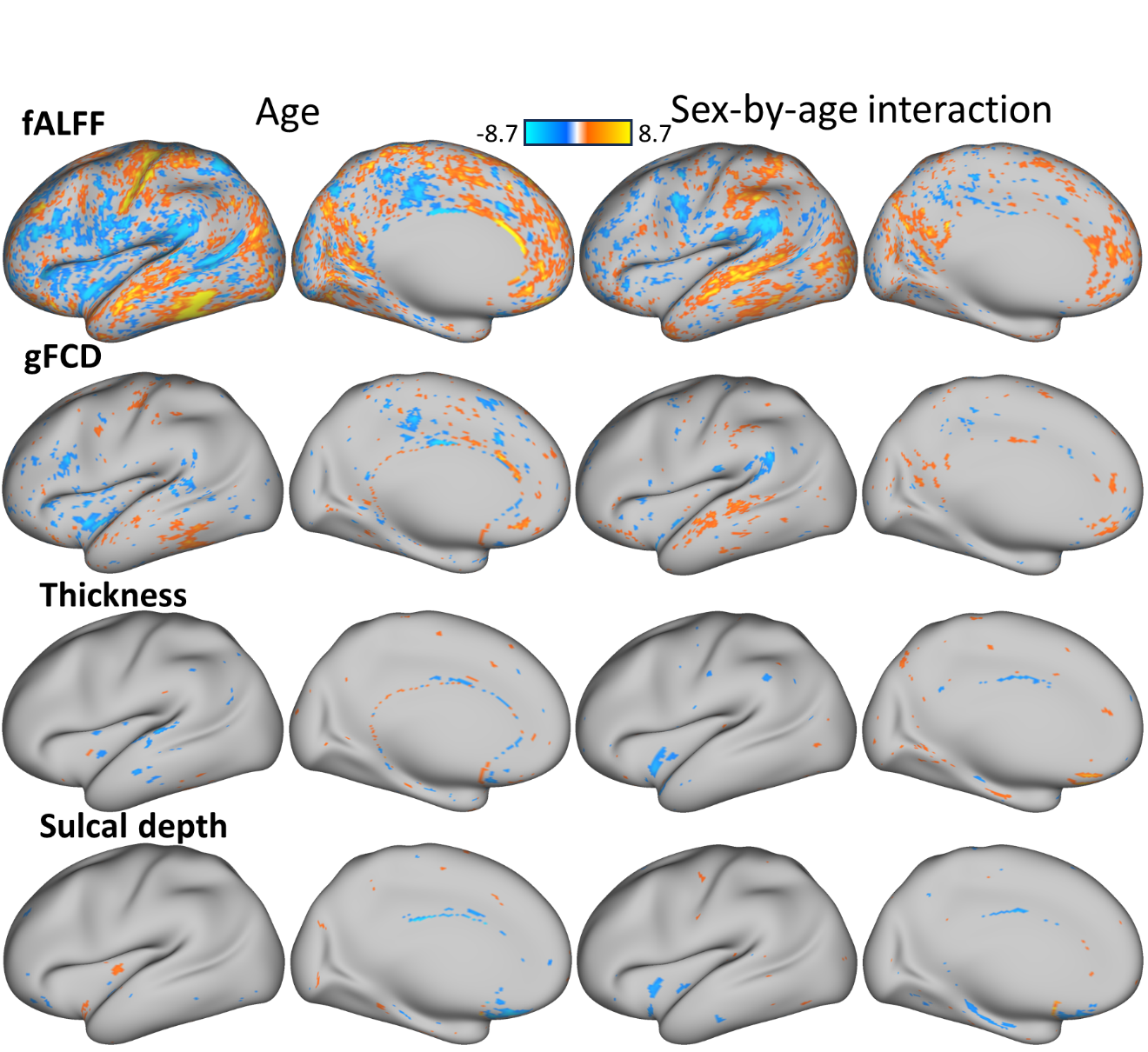


**Fig S4. Asymmetry & development.** T-score maps for the effect of age (left panels) and its interaction with sex (right panels) on the asymmetry index, Δ, for the fractional amplitude of low-frequency fluctuations (fALFF), global functional connectivity density (gFCD), cortical thickness, and sulcal depth, superimposed on lateral and medial views of the left cerebral hemisphere. A P_FDR_<0.05 corrected threshold is used for display. Sample: 8,721 children and 450 adolescents.


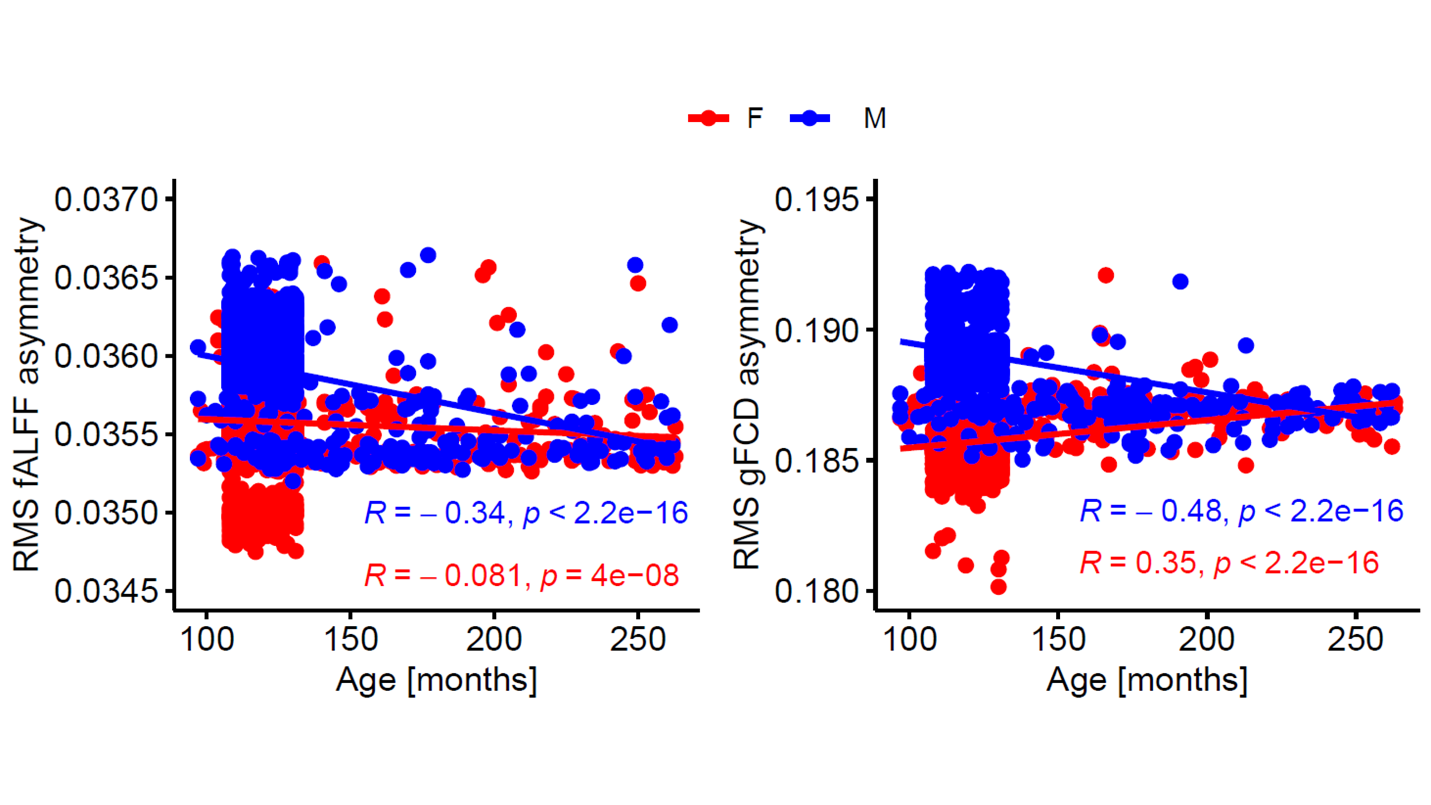


**Fig S5. Age-by-sex interaction effect on functional asymmetry**. Whole-brain root-mean square (RMS) values of fractional amplitude of low-frequency fluctuations (fALFF; left) and global functional connectivity density (gFCD; right) as a function of age for 4,694 boys and 4,477 girls. Sample: 8,721 ABCD children and 450 HCP-D adolescents.


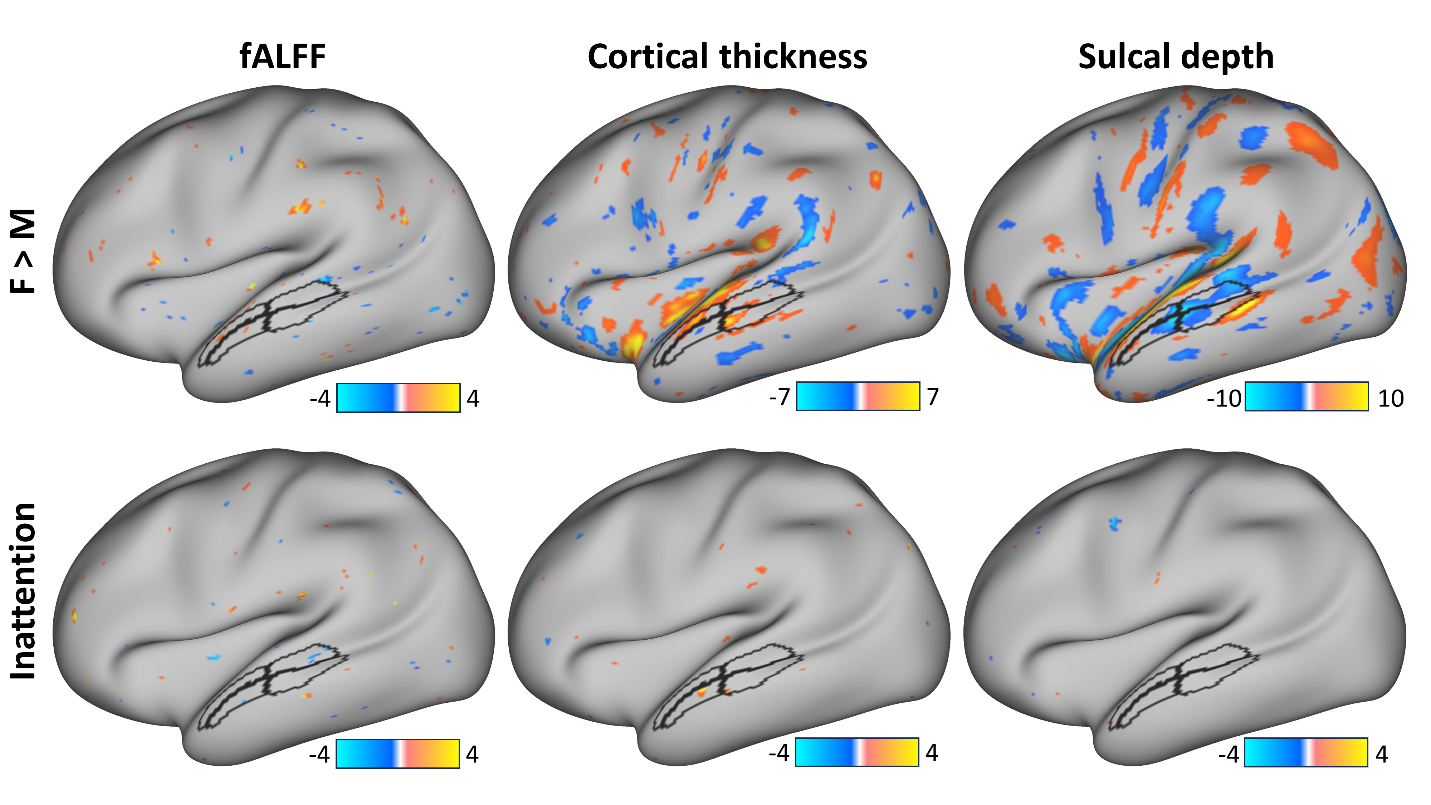


**Fig S6. Additional effects on asymmetry metrics.** Statistical effects (t-score) of sex (top row) and inattention (bottom row) on the asymmetries of the fractional amplitude of low-frequency fluctuations (fALFF), cortical thickness, and sulcal depth overlaid on a lateral view of the left cortical hemisphere. Sample: 4,238 girls (F) than 4,483 boys (M). Black lines are contours of four regions-of-interest in the superior temporal sulcus.


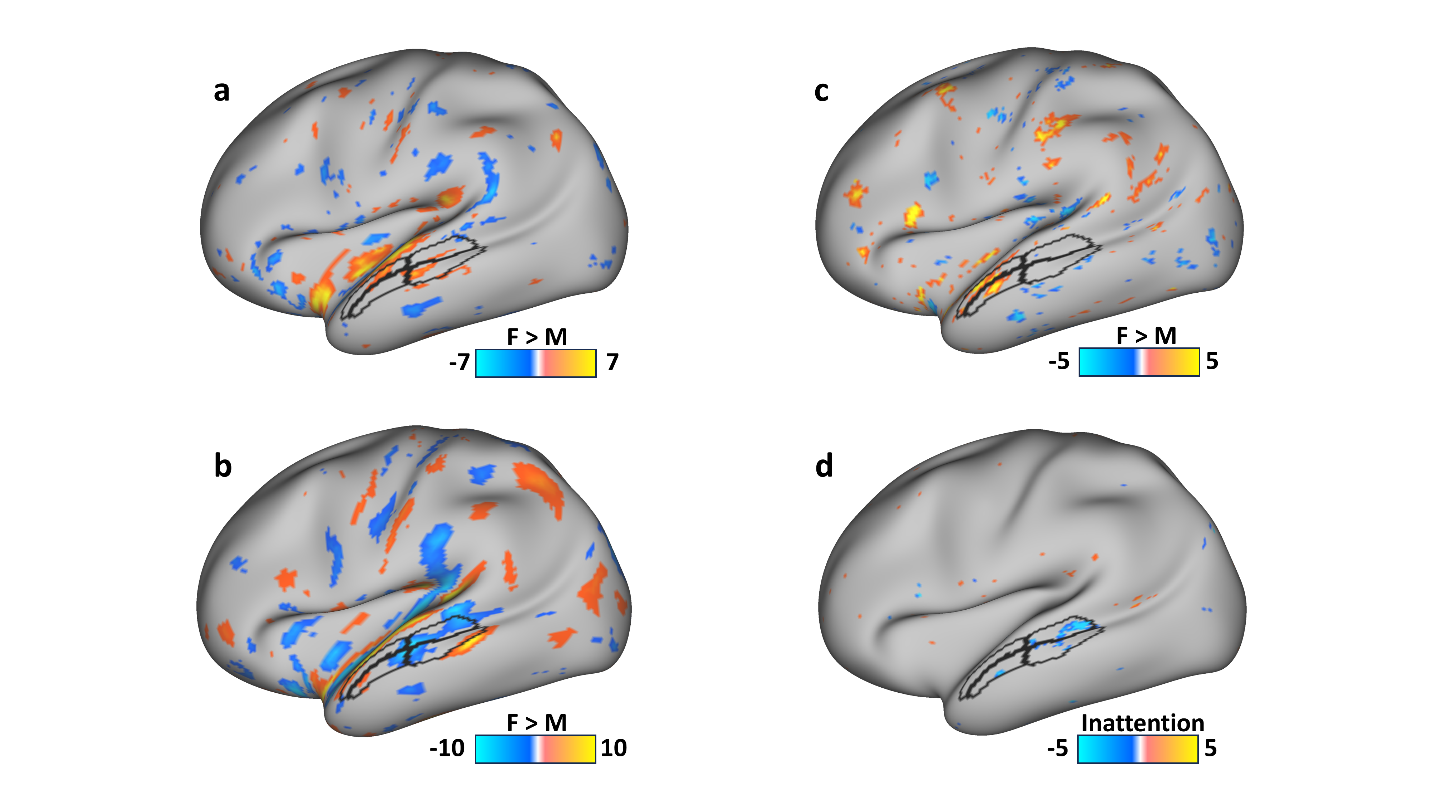


**Fig S7. Effects of sex and inattention on brain asymmetry in right-handed children.** Statistical differences (t-score) in the asymmetry of cortical thickness (**a**) and sulcal depth (**b**) overlaid on a lateral view of the left cortical hemisphere between left-handed girls (F; n=3,432) and boys (M; n=3,569). **c-d**) ANCOVA T-score maps showing the higher leftward asymmetry of gFCD in the anterior part of the superior temporal sulcus for girls than boys (**c**), and the association between higher inattention and lower leftward gFCD asymmetry in STS (**d**). A P_FDR_<0.05 threshold was used for display.


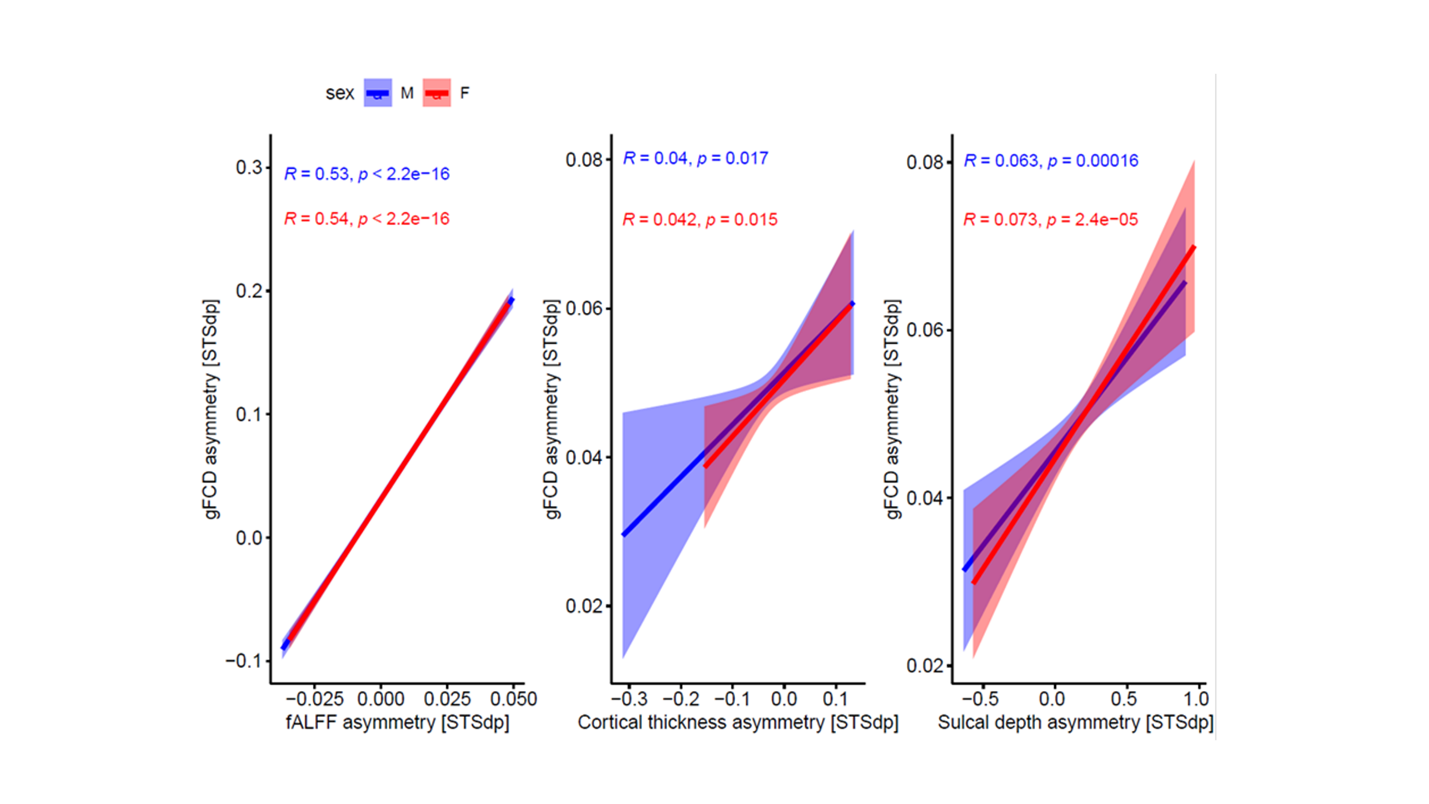
**Fig S8. Association between asymmetry metrics.** In the dorsal posterior part of the superior temporal sulcus (dpSTS) region-of-interest, the asymmetry of the global functional connectivity density (gFCD) was linearly associated with asymmetries in the amplitude of low-frequency fluctuations (fALFF), cortical thickness, and sulcal depth. Sample: 4,238 girls (F) than 4,483 boys (M).


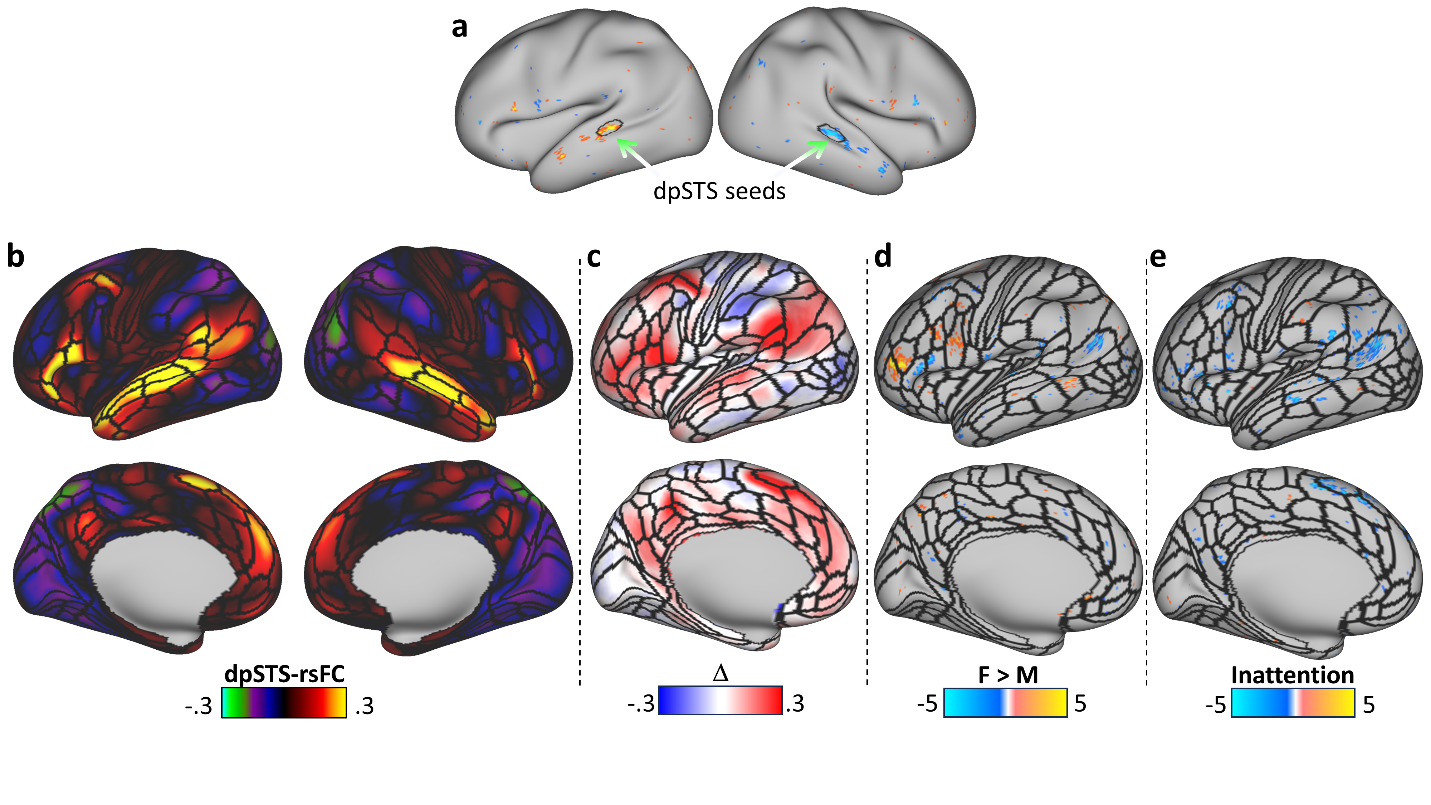


**Fig S9. dpSTS network in right-handers.** **a**) A bilateral seed in the dorsal posterior region of the superior temporal sulcus (dpSTS), which had lower asymmetry of global functional connectivity density with increased inattention scores, was used to map the resting-state functional connectivity (rsFC) using seed-vertex correlation analyses. **b**) dpSTS connectivity mapped into language network regions. **c**) dpSTS-rsFC asymmetry, Δ, mapped into the left cerebral hemisphere showing the strong leftward lateralization (red) of language network connectivity in PLS and the inferior frontal cortex. T-score maps reflecting statistically significant differences in Δ between 3,432 girls (F) and 3,569 boys (M; **d**) as well as associations with inattention scores and Δ (**e**), overlaid on the left cerebral hemisphere using a P_FDR_<0.05 corrected threshold. Black lines are contours of a multi-modal parcellation of the left cerebral cortex [9]. Statistical model: ANCOVA.


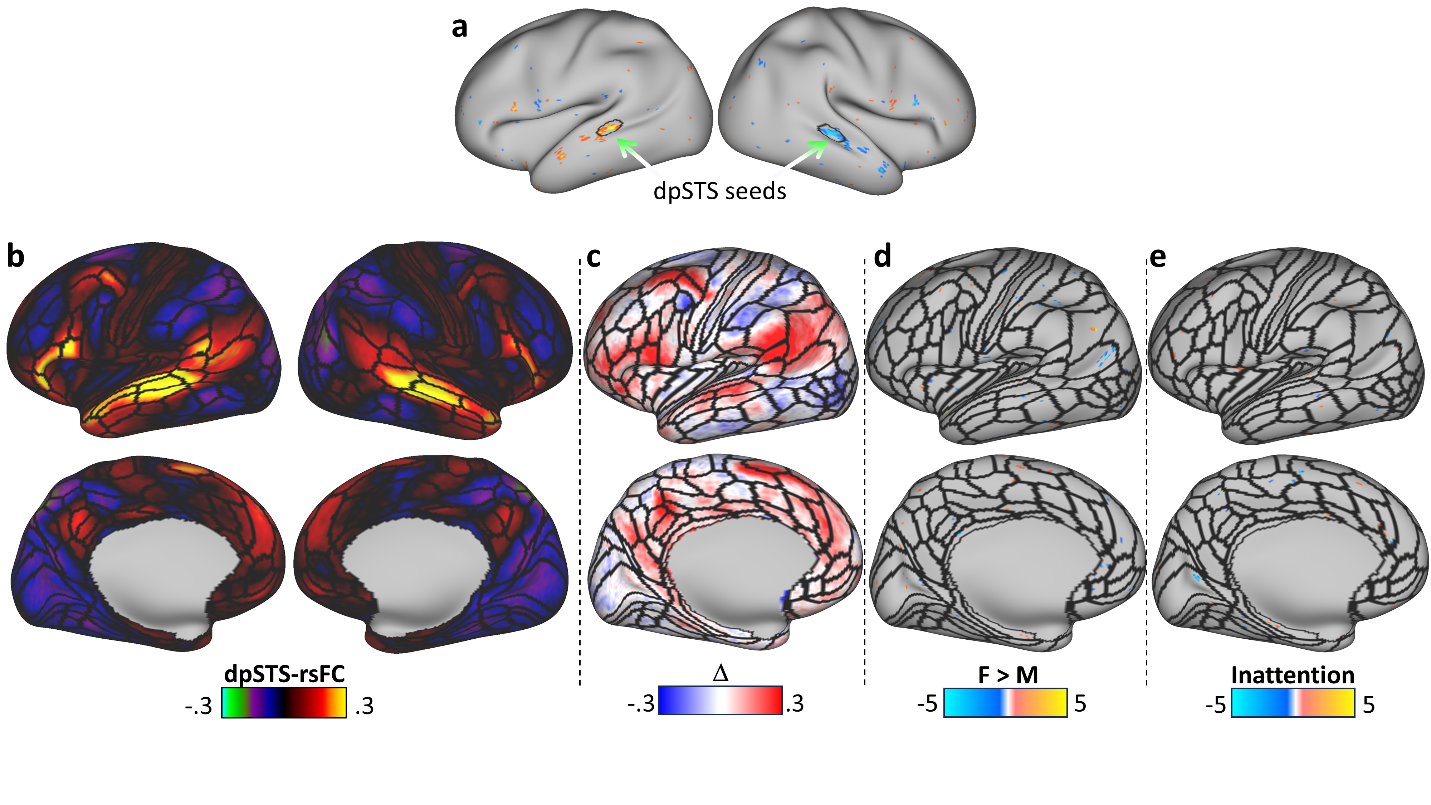


**Fig S10. dpSTS network in left-handers.** **a**) A bilateral seed in the dorsal posterior region of the superior temporal sulcus (dpSTS), which had lower asymmetry of global functional connectivity density with increased inattention scores, was used to map the resting-state functional connectivity (rsFC) using seed-vertex correlation analyses. **b**) dpSTS connectivity mapped into language network regions. **c**) dpSTS-rsFC asymmetry, Δ, mapped into the left cerebral hemisphere showing the strong leftward lateralization (red) of language network connectivity in PLS and inferior frontal cortex. T-score maps reflecting statistically significant differences in Δ between 273 girls (F) and 362 boys (M) left-handers (**d**) as well as associations with inattention scores and Δ (**e**), overlaid on the left cerebral hemisphere using a P_FDR_<0.05 corrected threshold. Black lines are contours of a multi-modal parcellation of the left cerebral cortex [9]. Statistical model: ANCOVA.

**
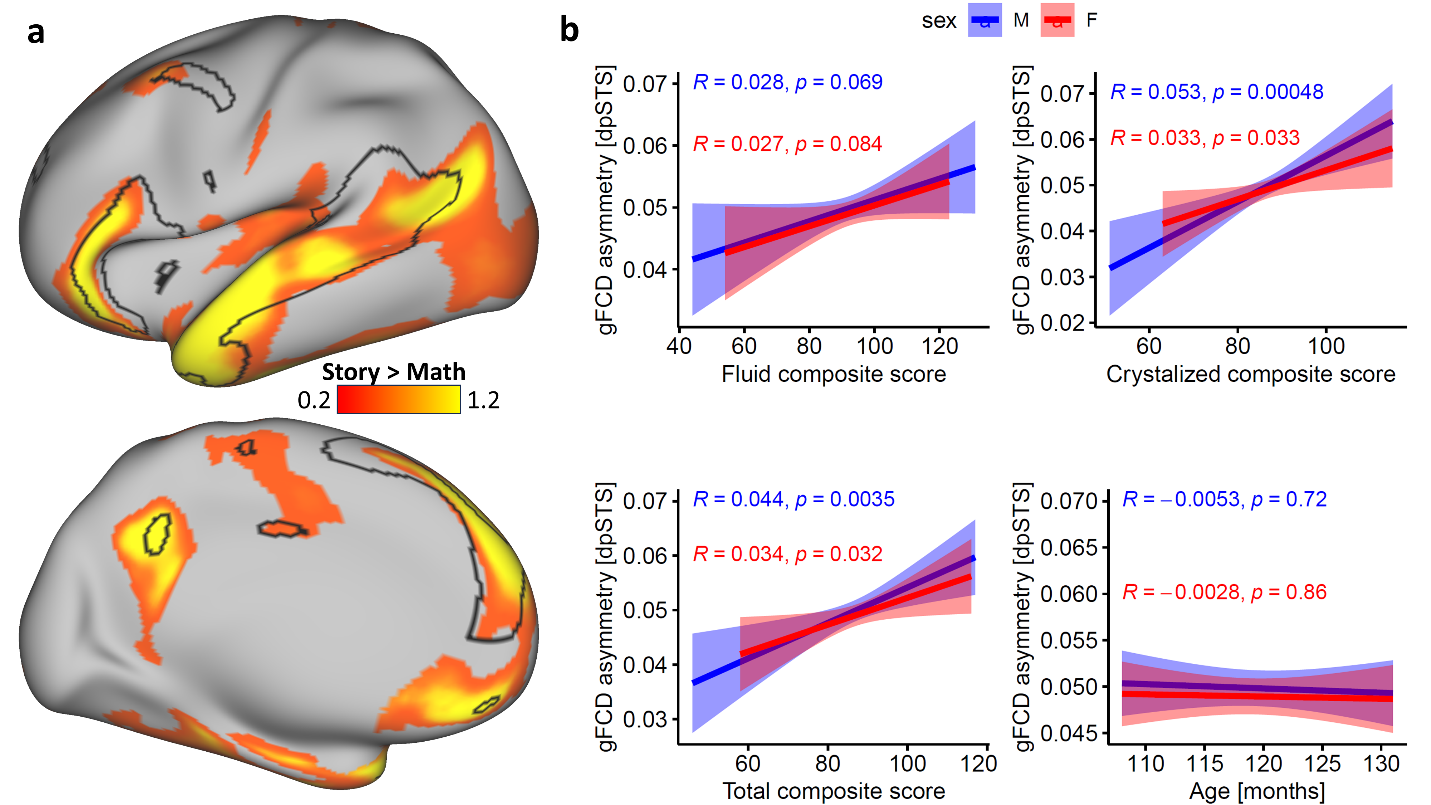
**

**Fig S11. Activation, asymmetry, and cognition.** **a**) Overlap between the dpSTS functional connectivity pattern (black contours) and the brain activation pattern (Cohen’s d map) to a language task contrasting “story” and “math” epochs across 997 healthy young adults from the HCP [9], overlaid on the left cerebral hemisphere using a display threshold P<0.00001. **b**) Scatter plots showing the linear associations of the asymmetry of global functional connectivity density (gFCD) in the dorsal posterior part of the superior temporal sulcus (dpSTS) with fluid, crystalized, and total composite scores, and the lack of associations with chronological age for 4,238 girls (F) and 4,483 boys (M) from the ABCD study; numeric labels reflect 2-sided Pearson correlations.


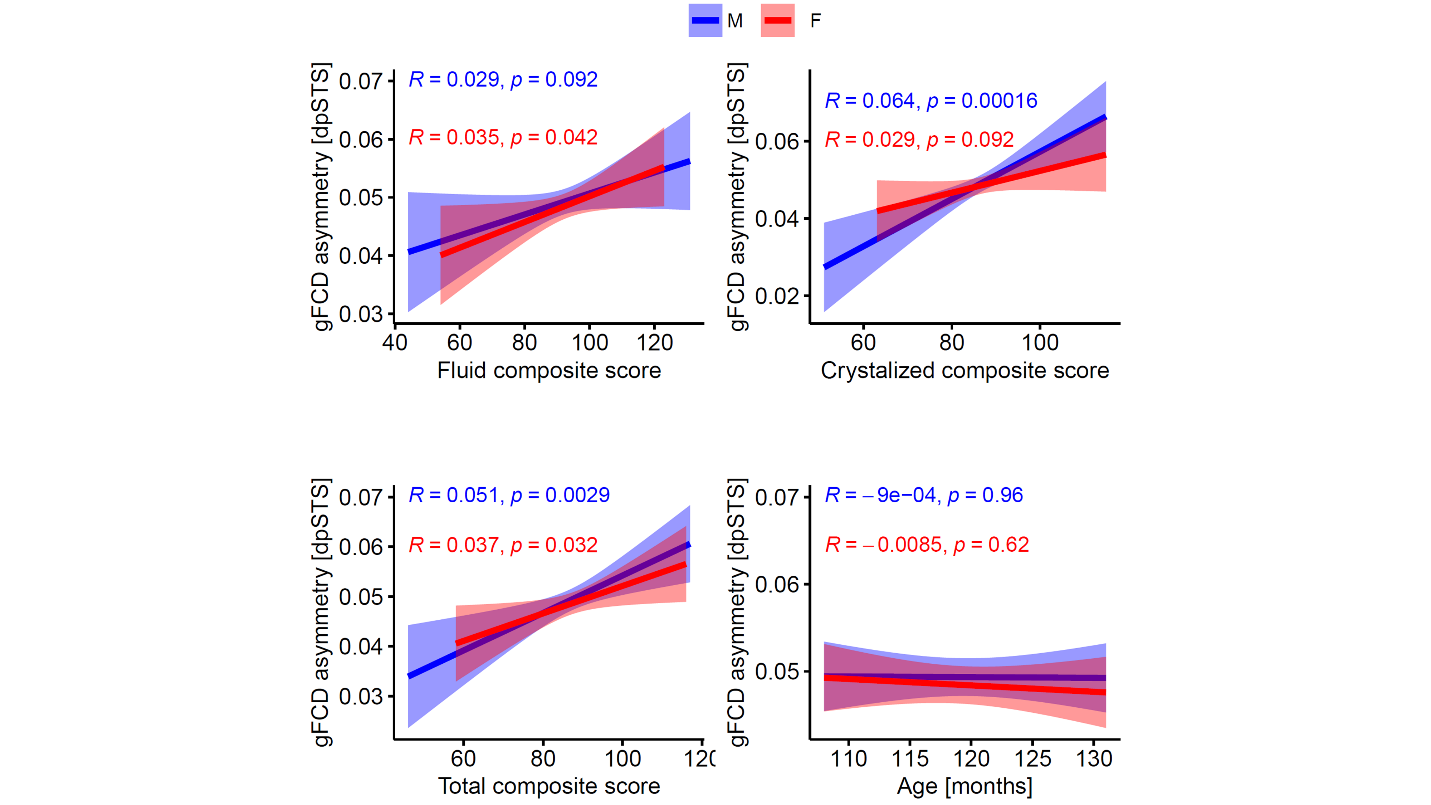


**Fig S12. Asymmetry and cognition in right handers.** Scatter plots showing the linear associations of the asymmetry of global functional connectivity density (gFCD) in the dorsal posterior part of the superior temporal sulcus (dpSTS) with fluid, crystalized, and total composite scores, and the lack of associations with chronological age for 3,432 girls (F) and 3,569 boys (M); numeric labels reflect 2-sided Pearson correlations.

**Table S1**: Regions-of-interest (ROIs) showing significant effects of inattention or sex on the asymmetry of cortical thickness, averaged within cortical partitions defined in the multimodal parcellation of the human cerebral cortex [9].

|  |  |  | Inattention | | Sex | |
| --- | --- | --- | --- | --- | --- | --- |
| ROI label | **Description** | **Cortical thickness**  **Asymmetry** | F | ges | F | ges |
| 33pr | Posterior rostral part of BA33 | 0.044 | - | - | 15.3 | 0.002 |
| 47s | Superior part of BA47 | -0.011 | - | - | 11 | 0.001 |
| 7m | Medial part of BA7 | -0.012 | - | - | 8.4 | 0.001 |
| A1 | Primary auditory cortex | -0.014 | - | - | 18 | 0.002 |
| A4 | Auditory 4 complex | -0.026 | - | - | 22.7 | 0.003 |
| A5 | Auditory 5 complex | -0.018 | - | - | 14.9 | 0.002 |
| IP2 | Area intraparietal 2 | 0.003 | - | - | 9.5 | 0.001 |
| LO2 | Area lateral occipital 2 | -0.019 | - | - | 10.5 | 0.001 |
| OFC | Orbitofrontal cortex | 0.001 | - | - | 12.1 | 0.002 |
| p24 | Posterior part of BA24 | -0.018 | - | - | 16.1 | 0.002 |
| PBelt | ParaBelt complex | -0.02 | - | - | 12.4 | 0.002 |
| PFcm | Parietal opercular area | 0.003 | - | - | 21 | 0.003 |
| PHA1 | Peri-hippocampal area 1 | 0.005 | - | - | 15.5 | 0.002 |
| PI | Para-insular area | 0.004 | - | - | 13.4 | 0.002 |
| PoI1 | Area posterior insular 1 | -0.019 | - | - | 13 | 0.002 |
| daSTS | Dorsal anterior part of STS | -0.013 | - | - | 32.8 | 0.004 |
| vaSTS | Ventral anterior part of STS | 0.009 | - | - | 9.5 | 0.001 |
| TA2 | Temporo-auditory area 2 | 0.004 | - | - | 23.5 | 0.003 |
| v23ab | Ventral part of BA23 a+b | -0.005 | - | - | 17.4 | 0.002 |
| V3B | Dorsal stream visual cortex | -0.005 | - | - | 16 | 0.002 |
| V4t | Posterior temporal visual area 4 | 0.004 | - | - | 12.2 | 0.002 |
| V6 | Parieto-occipital visual area | 0.003 | - | - | 13.9 | 0.002 |
| VVC | Ventral visual complex | -0.005 | - | - | 13 | 0.002 |

Statistical model: ANCOVA type 3 with 6 covariates: age, brain volume, cognitive performance, family income, scanner manufacturer, and remaining mean framewise displacement. Sample: 4,238 girls and 4,483 boys. ges: generalized eta square. BA: Brodmann area; STS: Superior temporal sulcus.

**Table S2**: Regions-of-interest (ROIs) showing significant effects of inattention or sex on the asymmetry of sulcal depth, averaged within cortical partitions defined in the multimodal parcellation of the human cerebral cortex [9].

|  |  |  | Inattention | | Sex | |
| --- | --- | --- | --- | --- | --- | --- |
| ROI label | **Description** | **Sulcal depth Asymmetry** | F | ges | F | ges |
| 1 | BA1 | -0.029 | - | - | 23.9 | 0.003 |
| 52 | BA52 | 0.052 | - | - | 80.7 | 0.01 |
| 10pp | Polar prefrontal part of BA10 | -0.04 | - | - | 7.5 | 0.001 |
| 31pv | Posterior ventral part of BA31 | -0.109 | - | - | 9.4 | 0.001 |
| 47l | Lateral part of BA47 | 0.003 | - | - | 14.1 | 0.002 |
| 47s | Superior part of BA47 | 0.101 | - | - | 29.7 | 0.004 |
| 7PC | Parietal cortex area 7 | -0.076 | - | - | 10.6 | 0.001 |
| 8BM | Medial part of BA8 | 0.117 | - | - | 7.6 | 0.001 |
| A4 | Auditory 4 complex | -0.234 | - | - | 42.5 | 0.005 |
| AAIC | Anterior agranular insular complex | -0.141 | - | - | 10.2 | 0.001 |
| EC | Entorhinal cortex | 0.03 | - | - | 7.1 | 0.001 |
| IPS1 | Intraparietal sulcus area 1 | -0.09 | - | - | 11.5 | 0.001 |
| LBelt | Lateral Belt Complex | -0.172 | - | - | 16.7 | 0.002 |
| LO3 | Area lateral occipital 3 | -0.117 | - | - | 7 | 0.001 |
| MT | Middle temporal area | 0.337 | - | - | 11.5 | 0.001 |
| OP1 | Opercular area 1 | -0.185 | - | - | 35.2 | 0.005 |
| OP2-3 | Opercular area 2-3 | -0.018 | - | - | 7.1 | 0.001 |
| p47r | Posterior rostral part of BA47 | 0.022 | - | - | 33.1 | 0.004 |
| PBelt | ParaBelt complex | -0.204 | - | - | 17.1 | 0.002 |
| PeEc | Perirhinal ectorhinal cortex | -0.063 | - | - | 23.7 | 0.003 |
| PFop | Opercular part of BA40 | 0.074 | - | - | 9.8 | 0.001 |
| PHA1 | Peri-hippocampal area 1 | 0.163 | - | - | 18.8 | 0.002 |
| PHA3 | Peri-hippocampal area 3 | 0.001 | - | - | 7.5 | 0.001 |
| PI | Para-insular area | 0.231 | - | - | 72.4 | 0.009 |
| POS1 | Parieto-occipital sulcus area 1 | 0.32 | - | - | 7.5 | 0.001 |
| RI | Retroinsular cortex | -0.049 | - | - | 34.5 | 0.004 |
| s32 | Superior part of BA32 | -0.252 | - | - | 29.5 | 0.004 |
| STGa | Anterior part of the superior temporal gyrus | -0.205 | - | - | 22.6 | 0.003 |
| dpSTS | Dorsal posterior part of STS | 0.156 | - | - | 40.9 | 0.005 |
| vpSTS | Ventral posterior part of STS | 0.104 | - | - | 19.4 | 0.002 |
| TPOJ1 | Temporo-parietal-occipital junction area 1 | 0.12 | - | - | 15.2 | 0.002 |
| TPOJ3 | Temporo-parietal-occipital junction area 3 | 0.216 | - | - | 10.5 | 0.001 |
| V1 | Primary visual cortex | -0.052 | - | - | 8 | 0.001 |
| VMV1 | Ventromedial visual area 1 | -0.121 | - | - | 8.2 | 0.001 |
| VMV2 | Ventromedial visual area 2 | 0.064 | - | - | 8.8 | 0.001 |
| VVC | Ventral visual complex | -0.024 | - | - | 7 | 0.001 |

Statistical model: ANCOVA type 3 with 6 covariates: age, brain volume, cognitive performance, family income, scanner manufacturer, and remaining mean framewise displacement. Sample: 4,238 girls and 4,483 boys. ges: generalized eta square. BA: Brodmann area; STS: Superior temporal sulcus.

**Table S3**: Regions-of-interest (ROIs) showing significant effects of inattention or sex on the asymmetry of the global functional connectivity density (gFCD) averaged within cortical partitions defined in the multimodal parcellation of the human cerebral cortex [9].

|  |  |  | Inattention | | Sex | |
| --- | --- | --- | --- | --- | --- | --- |
| ROI label | **Description** | **gFCD Asymmetry** | F | ges | F | ges |
| 1 | BA1 | 0.011 | - | - | 8.6 | 0.001 |
| 44 | BA44 | 0.006 | - | - | 24.5 | 0.003 |
| 45 | BA45 | 0.016 | - | - | 9 | 0.001 |
| 23d | Dorsal part of BA23 | -0.02 | - | - | 9.6 | 0.001 |
| 55b | Medial part of the premotor cortex | 0.019 | - | - | 8.4 | 0.001 |
| AAIC | Anterior agranular insular complex | -0.022 | - | - | 14.9 | 0.002 |
| DVT | Dorsal visual transitional area | 0.011 | - | - | 11.1 | 0.001 |
| i6-8 | Transitional area of the inferior premotor cortex | -0.015 | - | - | 12.1 | 0.002 |
| PFcm | Parietal opercular area | -0.039 | - | - | 10.9 | 0.001 |
| PHA3 | Peri-hippocampal area 3 | -0.005 | - | - | 16.8 | 0.002 |
| PI | Para-insular area | -0.036 | - | - | 33.8 | 0.004 |
| ProS | Pro-striate cortex | -0.002 | - | - | 8.4 | 0.001 |
| PSL | Peri sylvian language area | 0.01 | - | - | 10.5 | 0.001 |
| dpSTS | Dorsal posterior part of STS | 0.049 | 17.9 | 0.002 | 14.9 | 0.002 |
| TA2 | Temporo-auditory area 2 | -0.009 | - | - | 12.2 | 0.002 |
| TPOJ2 | Temporo-parietal-occipital junction area 2 | 0.013 | - | - | 16.6 | 0.002 |
| V4t | Ventral V4 transitional area | 0.047 | - | - | 17.9 | 0.002 |

Statistical model: ANCOVA type 3 with 6 covariates: age, brain volume, cognitive performance, family income, scanner manufacturer, and remaining mean framewise displacement. Sample: 4,238 girls and 4,483 boys. ges: generalized eta square. BA: Brodmann area; STS: Superior temporal sulcus.

**Table S4**: Regions-of-interest (ROIs) showing significant effects of inattention or sex on the asymmetry of the fractional amplitude of low-frequency fluctuations (fALFF) averaged within cortical partitions defined in the multimodal parcellation of the human cerebral cortex [9].

|  |  |  | Inattention | | Sex | |
| --- | --- | --- | --- | --- | --- | --- |
| ROI label | **Description** | **fALFF Asymmetry** | F | ges | F | ges |
| dpSTS | Dorsal posterior part of STS | 0.006 | - | - | 10.1 | 0.001 |
| V4 | V4 visual area | 0.002 | - | - | 11 | 0.001 |
| V4t | Posterior temporal visual area 4 | 0.005 | - | - | 14.7 | 0.002 |
| V8 | V8 visual area | 0.003 | - | - | 10.9 | 0.001 |

Statistical model: ANCOVA type 3 with 6 covariates: age, brain volume, cognitive performance, family income, scanner manufacturer, and remaining mean framewise displacement. Sample: 4,238 girls and 4,483 boys. ges: generalized eta square. BA: Brodmann area; STS: superior temporal sulcus.

**Table S5**: Regions-of-interest (ROIs) showing significant effects of inattention or sex on the asymmetry of the functional connectivity of the dorsal posterior part of the STS (dpSTS-rsFC), averaged within cortical partitions defined in the multimodal parcellation of the human cerebral cortex [9].

|  |  |  | Inattention | | Sex | |
| --- | --- | --- | --- | --- | --- | --- |
| ROI label | **Description** | **dpSTS-rsFC Asymmetry** | F | ges | F | ges |
| 44 | BA44 | 0.05 | 10.4 | 0.001 | - | - |
| 45 | BA45 | 0.041 | 11.9 | 0.002 | - | - |
| 10pp | Polar prefrontal part of BA10 | 0.011 | 8.9 | 0.001 | - | - |
| 31a | Anterior part of BA31 | 0.032 | 11.8 | 0.002 | - | - |
| 47s | Superior part of BA47 | 0.021 | 12.5 | 0.002 | - | - |
| 8Av | Anterior ventral part of BA8 | 0.036 | 9.1 | 0.001 | - | - |
| 8BM | Medial part of BA8 | 0.033 | 8.6 | 0.001 | - | - |
| 8C | Anterior ventral part of BA8 | 0.011 | 8 | 0.001 | - | - |
| 9m | Medial anterior part of BA9 | 0.026 | - | - | 12.5 | 0.002 |
| 9p | Posterior part of BA9 | 0.017 | 13.1 | 0.002 | - | - |
| a10p | Anterior part of posterior BA10 | 0.019 | 15.3 | 0.002 | - | - |
| a47r | Anterior rostral part of BA47 | 0.013 | 11.2 | 0.001 | - | - |
| AVI | Anterior ventral Insular area | 0.021 | 13.3 | 0.002 | - | - |
| FOP5 | Area frontal opercular 5 | 0.025 | 13.9 | 0.002 | - | - |
| IFSa | Anterior inferior frontal sulcus | 0.026 | - | - | 19.1 | 0.002 |
| PFcm | Parietal opercular area | 0.018 | 10.4 | 0.001 | - | - |
| PFm | Medial part of BA40 | 0.031 | 23.2 | 0.003 | - | - |
| PGi | Inferior part of the IPC | 0.03 | 19.3 | 0.002 | - | - |
| PGs | Superior part of the IPC | 0.026 | 18 | 0.002 | - | - |
| PIT | Posterior inferior temporal area | 0.001 | - | - | 11.7 | 0.002 |
| POS2 | Parieto-occipital sulcus area 2 | 0.021 | 10.8 | 0.001 | - | - |
| SFL | Superior frontal language area | 0.038 | 22.8 | 0.003 | - | - |
| dpSTS | Dorsal posterior part of STS | 0.08 | 16.4 | 0.002 | - | - |
| vpSTS | Ventral posterior part of STS | 0.019 | 10.3 | 0.001 | - | - |
| STV | Superior temporal visual area | 0.036 | 9 | 0.001 | - | - |
| TGd | Dorsal part of the temporal pole | 0.017 | - | - | 13.5 | 0.002 |
| TPOJ1 | Temporo-parietal-occipital junction area 1 | 0.015 | 10.6 | 0.001 | - | - |

Statistical model: ANCOVA type 3 with 6 covariates: age, brain volume, cognitive performance, family income, scanner manufacturer, and remaining mean framewise displacement. Sample: 4,238 girls and 4,483 boys. ges: generalized eta square. BA: Brodmann area; STS superior temporal sulcus; IPC: inferior parietal cortex.

**Table S6**: Percentage of variance explained by additive genetic factors (A), shared environmental factors (C), and non-shared environmental factors (E), degrees of freedom (df), and Bayesian (BIC) and Akaike (AIC) information criteria for behavioral and brain asymmetry metrics.

| Behavioral | A  [% variance] | C  [% variance] | E  [% variance] | df | BIC | AIC |
| --- | --- | --- | --- | --- | --- | --- |
| Fluid composite | 34±5*** | 22±4** | 44±2*** | 1388 | 10152 | 10157 |
| Crystalized composite | 61±5*** | 10±4* | 29±1*** | 1395 | 8515 | 8521 |
| Total composite | 52±5*** | 18±4** | 30±1*** | 1389 | 9377 | 9383 |
| Inattention score | 61±3*** | n.s. | 39±2*** | 1405 | 6927 | 6932 |
| Brain asymmetry |  |  |  |  |  |  |
| gFCD | 16±3*** | n.s. | 84±3*** | 1408 | -3581 | -3575 |
| fALFF | 8±3** | n.s. | 92±3*** | 1406 | -8729 | -8723 |
| Sulcal depth | 23±3** | n.s. | 77±3*** | 1408 | -630 | -624 |
| Cortical thickness | 5±3* | n.s. | 95±3*** | 1408 | -5351 | -5345 |

ACE model. p-value: <0.05(*); <0.001(**); or <1E-05(***). Estimated from 304 monozygotic twin pairs and 424 dizygotic twin pairs.

**References**

1. Garavan H, Bartsch H, Conway K, Decastro A, Goldstein R, Heeringa S *et al.* Recruiting the ABCD sample: Design considerations and procedures. *Dev Cogn Neurosci* 2018; **32:** 16-22.

2. Thompson W, Barch D, Bjork J, Gonzalez R, Nagel B, Nixon S *et al.* The structure of cognition in 9 and 10 year-old children and associations with problem behaviors: Findings from the ABCD study’s baseline neurocognitive battery. *Dev Cogn Neurosci* 2019; **36:** 100606.

3. Jernigan T, Brown S. Introduction. *Dev Cogn Neurosci* 2018; **32:** 1-3.

4. Karcher N, Barch D, Avenevoli S, Savill M, Huber R, Simon T *et al.* Assessment of the Prodromal Questionnaire-Brief Child Version for Measurement of Self-reported Psychoticlike Experiences in Childhood. *JAMA Psychiatry* 2018; **75**(8)**:** 853-861.

5. Zou Q, Zhu C, Yang Y, Zuo X, Long X, Cao Q *et al.* An improved approach to detection of amplitude of low-frequency fluctuation (ALFF) for resting-state fMRI: fractional ALFF. *J Neurosci Methods* 2008; **172**(1)**:** 137-141.

6. Tomasi D, Volkow N. Functional Connectivity Density Mapping. *Proc Natl Acad Sci U S A* 2010; **107**(21)**:** 9885-9890.

7. Tomasi D, Volkow N. Functional connectivity hubs in the human brain. *Neuroimage* 2011; **57**(3)**:** 908-917.

8. Tomasi D, Volkow N. Association between Functional Connectivity Hubs and Brain Networks. *Cereb Cortex* 2011; **21**(9)**:** 2003-2013.

9. Glasser M, Coalson T, Robinson E, Hacker C, Harwell J, Yacoub E *et al.* A multi-modal parcellation of human cerebral cortex. *Nature* 2016; **536**(7615)**:** 171-178.
